# Photoactuated Properties of Acetylene-Congeners Non-Metallic Dyes and Molecular Design for Solar Cells

**DOI:** 10.3390/ma11102027

**Published:** 2018-10-18

**Authors:** Nan Gao, Xiaochen Lin, Jinglin Liu, Yuanzuo Li, Yanhui Yang

**Affiliations:** 1 College of Science, Northeast Forestry University, Harbin 150040, China; nan_g@nefu.edu.cn; 2 Chemical Industry and Material College, Heilongjiang University, Harbin 150080, China; xiaochenlinhd@sohu.com; 3 Department of Physics, Jiamusi University, Jiamusi 154001, China; jinglinliujms@yeah.net; 4 Institute of Advanced Synthesis, School of Chemistry and Molecular Engineering, Jiangsu National Synergetic Innovation Center for Advanced Materials, Nanjing Tech University, Nanjing 211816, China; 5 School of Chemical and Biomedical Engineering, Nanyang Technological University, Singapore 639798, Singapore

**Keywords:** dye-sensitized solar cells (DSSC), Acetylene-congeners, DFT, TD-DFT, photoelectric characteristics, ICT

## Abstract

This paper theoretically simulated (using DFT and TD-DFT in N,N-dimethylformamide (DMF) solvent) the photodynamic properties of three non-metallic dye molecules with D-π-A_1_-π-A_2_ structure. The total photoelectric conversion efficiency (PCE) could be evaluated by the following parameters: the geometric structures, the electronic structures, and the absorption spectra, the analyses of charge difference density (CDD) and natural bond orbitals (NBO), the analyses of ionization potential (IP) and electron affinity (EA) from electronic contribution capacity, the reorganization energies ($\lambda_{h}$, $\lambda_{e}$, and $\lambda_{total}$), and the chemical reaction parameter (h, *ω*, $\omega^{-}$, and $\omega^{+}$) for intramolecular charge transfer (ICT) processing, the excited lifetime (τ) and the vertical dipole moment ($\mu_{normol}$). The $∆G^{inject}$, the $∆G_{dye}^{regen}$, the light harvesting efficiencies (LHE) and the excited lifetime (τ) were used to explain experimental $J_{SC}$. The experimental trend of $V_{OC}$ was explained by the calculation of $∆E_{CB}$ and $\mu_{normol}$. Moreover, the 15 dyes were designed by adding the electron-donor groups (–OH, –NH_2_, and –OCH_3_) and the electron-acceptor groups (–CF_3_, –F, and –CN) to the LS-387 molecular skeleton, which improved electronic contribution, intramolecular charge transfer (ICT), and optoelectronic performance.

## 1. Introduction

Since the beginning of the 21st century, environmental degradation and energy consumption have intensified. In order to realize the sustainable development of the environment and human society, it has become urgent to explore and develop new energy sources. Currently, silicon-based solar cells play a vital role in the field of energy with their excellent photoelectric conversion efficiency of about 26.6%. However, their disadvantages, such as high cost, non-renewable raw materials, difficulty in preparation and not easy to be improved, have limited their wide application. Since O’Regan and Grätzel in 1991 [1] reported the highly efficient dye-sensitized solar cells (DSSCs) based on Ru complex with photoelectric conversion efficiency (PCE) of 7.1–7.9%, more and more attention has been paid to DSSC due to its comparatively low cost and high efficiency [2,3,4,5]. DSSCs sensitized by free-metal organic dyes have been attracting attention by researchers for their clean and environment-friendly characteristics [6]. Particularly, the structural diversity and the simple synthetic routes of organic dye molecules provide the possibility to seek more competitive DSSC sensitizers [7]. For DSSCs, the core component is the sensitizing agent, which is divided into two kinds: metal-free organic dyes and metallic dyes [8]. Among them, organic dyes have the characteristics of low price, high extinction coefficient, adjustable structure, and light absorption characteristics by molecular design [9,10]. In order to achieve higher PCE, development of new structures, materials, and technologies has become important for researchers to improve conversion efficiency. From the viewpoint of molecular structures, donor-π bridge-acceptor (D-π-A) dyes have been widely used for non-metallic organic dye sensitizers; in addition, researchers have developed various molecular configurations (such as D-A-π-A, D-(π-A)_2_ and D-π-A_4_ etc.) to be used as the unit of the sensitizing agent for solar cells [11,12,13].

Non-metallic organic dyes with D-π-A-π-A structure have many advantages compared with the typical D-π-A structure [14,15,16], such as wider absorption spectra and improved intramolecular charge transfer ability. P Naik et al. reported non-metallic organic dyes with D-π-A-π-A structures (N1-3) from experiment and theory; dye N1 containing cyanoacetic acid as an acceptor unit showed a better PCE of 3.55% [14], and DFT calculations provided deeper understanding of the mechanism of experimental photovoltaic parameters from the viewpoint of charge separation between occupied and unoccupied molecular orbitals as well as matching simulated spectral data with experimental data. G Wang et al. reported an N,N-di-p-tolylaniline-based D-π-A_1_-π-A_2_ sensitizer XD_1_, obtaining a slightly higher PCE of 5.04% [15]. The influence of DFBT and DPP on the electron-density distribution and structural feature were revealed by DFT. Recently, the molecules associated with the D-π-A_1_-π-A_2_ were reported [16], in which LS-387 displayed a high PCE of 5.61%, with a higher short-circuit current ($J_{SC}$) of 13.26 $\mathrm{mA}/{\mathrm{cm}}^{2}$ and open-circuit voltage ($V_{OC}$) of 0.595 V; furthermore, effective intramolecular charge transfer (ICT) characteristics can be adjusted by changing the unit of donors. To understand the experimental micromechanism, we analyzed parameters of molecular geometric structure, electron absorption spectroscopy, frontier MOs, energy levels and gaps, charge-transfer, electron injection free energy, and dye regeneration characteristic for LS-385, LS-386, and LS-387 through DFT and TD-DFT theory [17,18,19]. Moreover, based on LS-387, a series of molecules was designed to detect how the modification of the donor and acceptor affects the $J_{SC}$ and $V_{OC}$. The main purpose was focused on D-π-A_1_-π-A_2_ organic materials, studying the relationship between structure and properties, and providing a design experience with specific functional groups.

## 2. Computational Methods

The quantum chemistry calculations were done using the GAUSSIAN09 software [20]. The ground state of three molecules (LS-385, LS-386, and LS-387) before and after absorbtion on ${\left({\mathrm{TiO}}_{2}\right)}_{9}$ were fully optimized in vacuum and N,N-dimethylformamide (DMF) solvent with DFT [21], using B3lyp/6–31G(d) [22]. Based on the geometrical optimization of ground state, the relative vibration frequencies were computed at the same level, showing the minimum value of the optimal potential energy surface. The molecular bond lengths and dihedral angles, the frontier MOs, the energy gaps, the injection and recombination parameters were calculated. The absorption and emission characteristics of the three dyes in vacuum and solvent were obtained with TD-DFT [23] by using the CAM-B3LYP [24] functional with the 6–31G(d) basis set. Natural bond orbitals (NBO) analysis [25] based on the difference in charge between the ground state and the excited state was simulated using the NBO 6.0 program [26]. Furthermore, the Multiwfn 3.4 program [27] combined with the VMD 1.9.3 program [28] was used to visually analyze electrostatic potential (ESP) and average local ionization energy (ALIE). Moreover, we also calculated the first static hyperpolarization $\beta_{tot}$ of three molecules [29].

It is well known that the efficiency of DSSCs can be calculated from the $V_{OC}$, $J_{SC}$, fill factor (FF) and the incident solar power on the cell ($P_{in}$). Calculated efficiency can be written as follows [30]:

(1) $$\eta \left(\%\right)=\frac{V_{OC}\times J_{SC}\times \mathrm{FF}}{P_{in}}\times 100\%$$

The fill factor (FF) is defined as the ratio of the maximum power that the battery can output to the theoretical maximum output power of the product of the $J_{SC}$ and $V_{OC}$:

(2) $$\mathrm{FF}=\frac{I_{m}\times V_{m}}{V_{OC}\times J_{SC}}$$

where $I_{m}$ and $V_{m}$ are the current and voltage corresponding to the maximum output power of the battery.

The $J_{SC}$ is an important representation of the PCE for DSSC, which can be expressed as [31]:

(3) $$J_{SC}=\underset{\lambda }{\int }\mathrm{IPCE}\left(\lambda \right)dy$$

where IPCE is the incident photon-to-electron conversion efficiency, which can be obtained by the following calculation formula:

(4) $$\mathrm{IPCE}=\mathrm{LHE}\left(\lambda \right)\phi_{inject}\eta_{collect}\eta_{reg}I_{s}$$

where LHE(λ) represents the light harvesting efficiency, and $\phi_{inject}$ indicates the electron injection efficiency, $\eta_{collect}$ is the charge collection efficiency, and $\eta_{reg}$ expresses the regeneration efficiency of dyes.

For particular DSSCs, the charge collection efficiency ($\eta_{collect}$) is only a negligible difference in the same semiconductor electrode (universal is ${\mathrm{TiO}}_{2}$). Therefore, the $J_{SC}$ is determined by the remaining three parameters: LHE, $\phi_{inject}$, and $\eta_{reg}$. The LHE can be expressed as [32,33]:

(5) $$\mathrm{LHE}=1-10^{-A}=1-10^{-f}$$

where *f* is the calculated oscillator strength.

## 3. Results and Discussion

### 3.1. Geometric Structures

**Figure 1:**
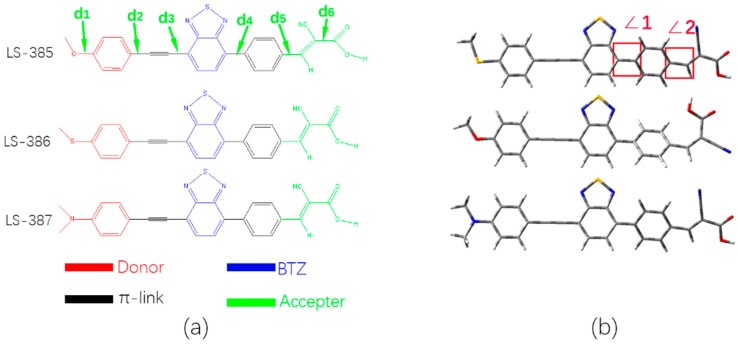
(**a**) shows molecular structures and names of LS-385, LS-386, and LS-387; (**b**) shows the ground-state optimized geometry by DFT calculations performed at the 6–31G(d) level.

The ground state geometries of three organic molecules were calculated by using DFT/B3lyp with 6–31G(d) basis set in vacuum and DMF solvent. As shown in Figure 1a, the three molecules have a similar acceptor and π-bridge based on the benzene ring and the auxiliary acceptors of benzothiadiazole (BTZ) units near the acetylene bridge; the only difference is that the donor unit has different atom of oxygen, sulfur, and nitrogen. Based on the similarity of the three molecules, we defined six bond lengths $d_{1}$ to $d_{6}$ (see Figure 1a). In vacuum, $d_{1}$ of three molecules shows a great difference due to the differences in the donor group (such as O, S, and N atoms), and the single bonds $d_{1}$ show a shorter bond length for C–O (1.360 Å) and C–N(1.379 Å) (see Table 1), respectively. Moreover, in solvent, the $d_{1}$ for the three molecules is less than that in vacuum. The DFT calculation shows that the relatively short bond length may be derived from the hybridization between $sp^{2}$ and $sp^{3}$ carbon [34], and the corresponding bond length value has a good correlation with the photoelectric properties. By comparing $d_{2}$ to $d_{6}$ in vacuum and solvent, LS-387 has a shorter bond length, and thus LS-387 has better molecular stability. However, there is no significant difference between LS-386 and LS-385.

The acetylene bridge plays a crucial role in the coplanarity between the benzothiadiazole (BTZ) and the donor group (see Figure 1b). The two dihedral angles ∠1 and ∠2 in vacuum and DMF solvent are listed in Table 1. However, due to the spatial repulsive force between the nitrogen atom and the hydrogen atom of the benzene ring in the BTZ structure, the dihedral angle (∠1) between BTZ and the adjacent phenyl group produces an angle of about 33° (See Table 1). For ∠2, compared with the vacuum, the dihedral angles in solvent are smaller, and LS-387 has the minimal dihedral angle (0.5°) compared with LS-385 and LS-386 in DMF solvent.

### 3.2. Electronic Structure

**Figure 2:**
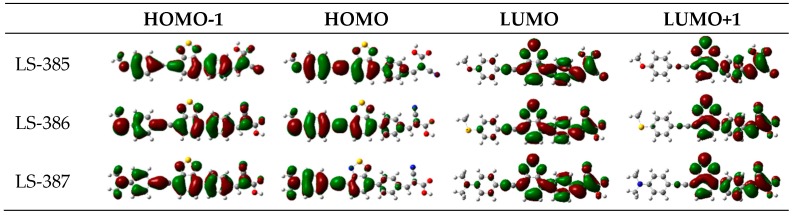
Frontier molecular orbital of LS-385, LS-386, and LS-387 calculated under B3LYP/6–31G(d) level.

**Figure 3:**
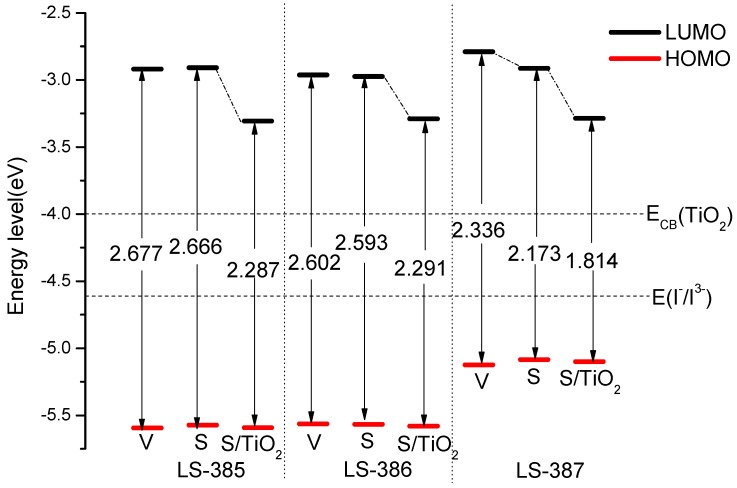
The molecules energy levels diagram of LS-385, LS-386, LS-387 (V, S, and S/${\mathrm{TiO}}_{2}$ representative in vacuum, in solvent and dyes/${\mathrm{TiO}}_{2}$ in solvent, respectively).

Electronic structure analysis gives the charge transfer characteristics. The energy level of HOMO, LUMO, and energy gaps (${∆}_{H}=\left|H-L\right|$) and electron density of the frontier MOs are the important parameter reflecting the electronic excitation and transition characteristics of the dyes, shown in Table S1 and Figure 2 and Figure 3. As shown in Figure 2, HOMO and LUMO belong to the π and $\pi^{*}$, respectively [35]. For LS-385 and LS-386, the electron density of HOMO is distributed on the D-π-$A_{1}$-π part; the electron density of LUMO resides in the π-$A_{1}$-π-$A_{2}$ part; and for LUMO+1, the electron density is the distribution on the $A_{1}$-π-$A_{2}$ part; most of the electrons are in the acceptor; and for the HOMO-1, the electron is distributed throughout the molecule. LS-385 and LS-386 exhibit a similar electron density distribution. For LS-387, the LUMO distribution is not significantly different from the other two molecules, but HOMO energies have a good aggregation on the donor, indicating that LS-387 has a better push-pull effect.

The driving force for electron injection and oxidation dye regeneration can be evaluated by the energy levels. As shown in Figure 3, the LUMO energy levels of three molecules are higher than the conduction band (CB) of TiO_2_ [$E_{CB}\left({\mathrm{TiO}}_{2}\right)$] of −4.0 eV, which facilitates electron injection from the excited dyes to the TiO_2_ electrode. The LUMO energy levels of LS-385, LS-386, and LS-387 are lower than that of the redox potential $I^{-}$/$I^{3-}$ (−4.60 eV [36,37]), which means that the electrolyte can release electrons into the oxidative dye. From Table S1, the HOMO energies of the three molecules in vacuum can be arranged as LS-387 (−5.125 eV) > LS-386 (−5.564 eV) > LS-385 (−5.595 eV), it is probably because the N atom in LS-387 donor is effective in reducing the HOMO level. The LUMO energies are in following order: LS-387 (−2.789 eV) > LS-385 (−2.918 eV) > LS-386 (−2.962 eV), it can be concluded that both HOMO and LUMO of LS-387 are greater than for other molecules. Higher HOMO energy can result in higher electron donation capabilities, meaning that LS-387 has strong electronic donation capabilities. In solvent, the HOMO and LUMO of LS-385 and LS-386 do not show obvious changes compared with vacuum (see in Figure 3). While for LS-387 in solvent, the HOMO is greater than that in vacuum, and the LUMO is less than that in vacuum.

The HOMO and LUMO energy levels after adsorption on titanium dioxide are shown in Figure 3. The HOMO energy of LS-387/s and LS-387/s + ${\mathrm{TiO}}_{2}$ are −5.085 eV and −5.100 eV (see Table S1), respectively. It is obvious that the HOMO has changed slightly before and after adsorption onto ${\mathrm{TiO}}_{2}$. For LUMO, LS-387/s + ${\mathrm{TiO}}_{2}$ (−3.286 eV) is significantly higher than LS-387/s (−2.912 eV). A similar trend also occurs in the other two molecules. In addition, the energy gap also shows a downward trend compared with isolated molecules; their values are: LS-385/s + ${\mathrm{TiO}}_{2}$ (2.287 eV), LS-386/s + ${\mathrm{TiO}}_{2}$ (2.291 eV), and LS-387/s + ${\mathrm{TiO}}_{2}$ (1.814 eV).

**Figure 4:**
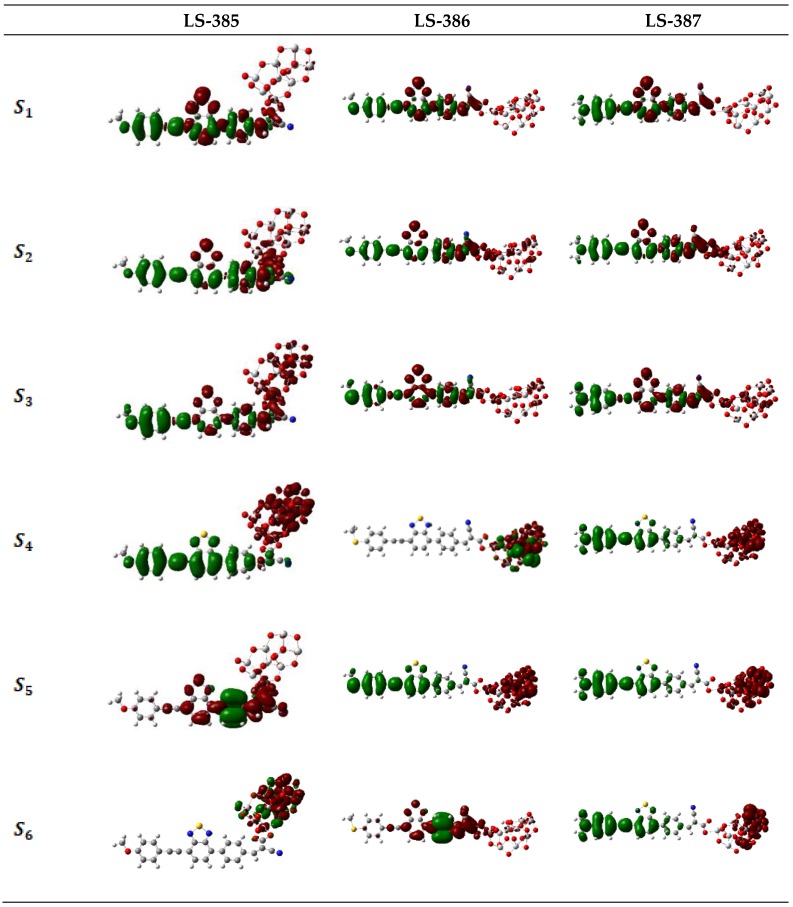
Charge difference density (CDD) of the selected excited state for dyes/${\left({\mathrm{TiO}}_{2}\right)}_{9}$ complexes in solvent. (Green and red stand for the hole and electron, respectively).

The charge difference density (CDD) of the three molecules was used to study the charge transfer characteristics (see Figure S1). The CDD map clearly shows the change of charge density between the ground state and the excited state during photo-excitation, [38,39], indicating the ICT direction. As shown in Figure S1, the electron density is mainly distributed in BTZ units and acceptor, and the hole density is mainly distributed in donor, π-bridge, and BTZ, therefore, CI is from donor to acceptor. Figure 4 shows the CDD of the dye and ${\mathrm{TiO}}_{2}$ complex model, which has a more obvious charge separation compared with the isolated dye molecules. As shown in Figure 4, for LS-385/${\mathrm{TiO}}_{2}$ and LS-386/${\mathrm{TiO}}_{2}$, the electron density is gradually transferred into ${\mathrm{TiO}}_{2}$ clusters with the increase of energy levels, and the hole density is gathered in the site of the donor. For LS-387/${\mathrm{TiO}}_{2}$, with the increase of the energy levels, the separation of electrons and holes become gradually obvious; for $S_{4}–S_{6}$ it seems that the electrons tend to be distributed in ${\mathrm{TiO}}_{2}$ clusters on one site, while the hole distribution is on the molecule near the site of the donor, thus enhancing the ICT characteristics of LS-387.

### 3.3. Electronic Absorption Spectra

Based on the geometry optimization of the ground-state, the excited states of the three dyes and dyes/${\mathrm{TiO}}_{2}$ were calculated based on TD-DFT/cam-B3lyp/6–31G(d) in vacuum and DMF solvent. As compared, a diffused basis set 6–31+G(d,p) was used to calculate $\lambda_{max}$ on the basis of optimization with the same basis set, and the values of $\lambda_{max}$ for LS-385, LS-386, LS-387, are 543.27 nm, 564.42 nm and 671.80 nm, respectively, which are greatly red-shifted in comparison with experiment (425 nm, 425 nm, and 475 nm). Therefore, the basis set 6–31G(d) was used in the following calculations due to the wide application and certain accuracy. As shown in Table 2, the maximum wavelength ($\lambda_{max}$) of the three molecules in vacuum can be arranged as follows: LS-387 (449.11 nm) > LS-386 (426.65 nm) > LS-385 (421.57 nm), and LS-387 has about 25 nm red-shift. In solvent, the $\lambda_{max}$ is in order: LS-387 (470.40 nm) > LS-386 (428.83 nm) ≈ LS-385 (428.47 nm), and LS-387 also has about 40 nm red-shift compared with LS-385 and LS-386, which is due to the fact that LS-387 has a smaller energy gap to exhibit a high molar extinction coefficient and produce more electrons under visible light. Meanwhile, the LS-387 also showed higher $V_{OC}$ in the experiment [16].

**Figure 5:**
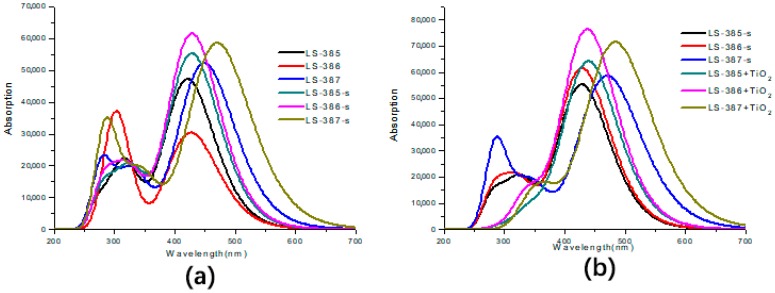
UV-Vis Absorption spectra of LS-385, LS-386, and LS-387 (where (**a**) represents in vacuum and (**b**) represents in DMF solvent).

As shown in Figure 5a, the UV-Vis absorption spectra of the three dye molecules in vacuum and solvent cover the near-ultraviolet and visible regions, and they all have distinct double absorption peaks. The highest absorption peak is due to the first excited state ($S_{1}$), and its electronic transition is from HOMO to LUMO, showing better ICT characteristics. For LS-385 in vacuum and solvent, the lower absorption peak (located near 325 nm) is mainly attributed to the second excited state ($S_{2}$), and the corresponding electronic transition is from HOMO to LUMO + 1 (*f* = 0.5094 and 0.4518 in vacuum and solvent, respectively). It can be seen from the similar charge distribution of LUMO and LUMO + 1 that the electron transfer pathway is similar to that of $S_{1}$. For LS-386 in solvent, the main absorption peak at 330 nm corresponds to the second excited state ($S_{2}$), it shows an electron transition from HOMO to LUMO + 1 (*f* = 0.4001); and for LS-387 in vacuum and solvent, the second absorption peak (near 340 nm) corresponds to a transition from HOMO→LUMO + 1 in the S2 (*f* = 0.4445 and 0.4431 in vacuum and solvent, respectively); similarly, the transition of this state is the same as $S_{1}$. In summary, $\lambda_{max}$ is mainly ICT derived from the $S_{1}$ excited state. Figure 5b shows the UV-Vis absorption spectra of three dyes after adsorbing on ${\mathrm{TiO}}_{2}$ cluster, and the absorption spectra of three dyes having red-shifted compared to isolated dyes. Moreover, the molar extinction coefficients of LS-386 and LS-387 have a marked increase of 7.64 × $10^{4}$ $M^{-1}{\mathrm{cm}}^{-1}$ and 7.14 × $10^{4}$ $M^{-1}{\mathrm{cm}}^{-1}$, respectively. Therefore, the absorption spectrum of dyes after adsorption has obviously changed, which can increase the ICT and the electron transfers into ${\mathrm{TiO}}_{2}$CB.

The analysis of natural bond orbitals (NBO) provides a deeper understanding of the optical excitation properties of dyes. As shown in Table 3, the difference in charge (∆q, from $S_{0}$ to $S_{1}$) of the three molecules at the donor group indicates that LS-387 and LS-386 have a strong electron-providing ability compared with LS-385. This is probably because oxygen atoms on the LS-385 donor have poor electron capacity. Compared with LS-385 and LS-386, the BTZ group of LS-387 sneaked into the electron collection of the receptor. Besides, ∆q on the acetylene bridge of LS-385 (−0.09), LS-386 (−0.08), and LS-387 (−0.086), provide an ICT channel. Also, the acceptor of ∆q shows the following: LS-387 (0.09) > LS-386 (0.08) ≈ LS-385 (0.08), which illustrates that LS-387 has a strong ability to accept electrons. As a result, LS-387 should stimulate more electron transfer in the optical excitation mechanism.

### 3.4. Analysis of Charge Injection Capability.

An important indicator for assessing injection capacity is ionization potential (IP) and electron affinity (EA) for holes and electrons, respectively [40,41,42,43]. IP represents the energy change of electron extraction or hole addition. EA means the change in energy of hole extraction or electron absorption [35]. As shown in Table S2, the IP of the three molecules in the order: LS-385(6.67 eV) > LS-386 (6.64 eV) > LS-387 (6.23 eV), and the smallest LS-387 has the better injection capacity. The EA of the three dyes is as follows: LS-387 (1.80 eV) < LS-385 (1.93 eV) < LS-386 (1.99 eV), and the higher the EA, the higher the electronic acceptability becomes [44]; so LS-386 has a greater ability to inject electrons. Nevertheless, the IP of the three dyes in solvent goes down compared with vacuum, and LS-387 in solvent has the lower IP of 4.92 eV, which also indicates that LS-387 is more advantageous for extracting electrons. The $E_{fund}=IP-EA$ is used to characterize the electronic contribution of the dye molecules. As shown in Table S2, the $E_{fund}$ of the three dyes in vacuum is as follows: LS-385 (4.74 eV) > LS-386 (4.65 eV) > LS-387 (4.43 eV), and in solvent the dye of LS-387 has a lower $E_{fund}$ of 1.76 eV, which is in agreement with the experiment [16]. The $E_{fund}$ is vacuum > solvent, therefore, the molecules in the DMF solvent produce a better electronic capability than in vacuum.

### 3.5. Analysis of Chemical Reaction Parameters

Another method for evaluating the charge transfer properties of sensitizers is the recombination energy [35], and the Marcus theory gives the rate formula [45]:

(6) $$K_{ET}=A\exp[-\lambda /4K_{B}T]$$

where *λ* is the recombination energy, *T* is the temperature, A is the electron coupling, and $K_{B}$ is the Boltzmann constant.

Quantitative methods provide a feasible method for studying charge transport in organic material systems [46] and for calculating hole and electron recombination energy ($\lambda_{h}$ and $\lambda_{e}$), which can be calculated [46]:

(7) $$\lambda_{h}=(E_{0}^{-}-E_{-})+(E_{0}^{-}-E_{0})$$

(8) $$\lambda_{e}=\left(E_{0}^{+}-E_{+}\right)+\left(E_{0}^{+}-E_{0}\right)$$

The above parameters can be obtained by optimizing the neutral molecular structure and the anion (cation) structure. As shown in Table 4, $\lambda_{h}$ of the three molecules in vacuum can be arranged: LS-385 (0.25 eV) > LS-386 (0.20 eV) > LS-387 (0.18 eV); and $\lambda_{e}$ can be arranged in the order: LS-386 (0.42 eV) > LS-385 (0.37 eV) > LS-387 (0.33 eV). Table 4 also shows the $\lambda_{h}$ and $\lambda_{e}$ in DMF solvent has a noticeable decrease, and LS-387 has a lower recombination energy in vacuum and solvent, which will produce better molecular charge transfer and thus better photoelectric performance.

Electrochemical parameters (such as chemical hardness h, electrophilic index *ω*, electron accepting power $\omega^{+}$, and electron donating ability $\omega^{-}$) are important factors affecting the efficiency of photovoltaic cells. The relevant calculated data are in Table 4. The parameter h represents the impedance for ICT [47]. Low chemical hardness is characterized by low ICT resistance, which in turn increases the acceptability of electrons [40]. Therefore, good dyes should have low h and higher $\omega^{+}$. As shown in Table 4, LS-387 (h = 2.22 eV) has a lower chemical hardness compared with that of LS-385 (h = 2.37 eV) and LS-386 (h = 2.32 eV), and the h of three molecules is: vacuum > solvent, indicating that LS-387 has a smaller ICT resistance in solvent. Moreover, LS-387 in solvent has a higher electron accepting power ($\omega^{+}$ = 7.36) than LS-385 ($\omega^{+}$ = 6.41) and LS-386 ($\omega^{+}$ = 6.56), which implies that LS-387 exhibits a higher electron withdrawing capacity through its receptor moiety. Taken the two parameters into account, it can be inferred that LS-387 will have higher ICT and PCE. The higher the electrophilic index (*ω*), the higher the stability of the dye becomes. In solvent, LS-387 has a higher electrophilicity index (*ω* = 9.27 eV) compared with that of LS-385 (*ω* = 8.41 eV) and LS-386 (*ω* = 8.57 eV), and the *ω* of three dyes is solvent > vacuum, which indicate LS-387 has a higher energetic stability. In order to obtain a large electron supply capacity, the hope is that the molecule has a lower electron donating energy. Table 4 shows that LS-387 in vacuum has a lower electron donating power ($\omega^{-}$ = 5.93 eV) compared with that of LS-385 ($\omega^{-}$ = 6.35 eV) and LS-386 ($\omega^{-}$ = 6.46 eV); however, in solvent, the three dyes have a higher $\omega^{-}$ compared with that in vacuum. Never the less, comprehensive consideration on the chemical reactivity parameters indicates that LS-387 in solvent has a better chemical reactivity parameter, resulting in a better photoelectric performance of LS-387 among the three dyes.

### 3.6. Performance of DSSCs Based on Dyes

The LHE is an important parameter to measure the performance of sensitizer and to evaluate $J_{SC}$. Table 5 shows LS-387 (0.9485) in vacuum has a higher LHE compared with LS-385 (0.9315) and LS-386 (0.8220). Moreover, in DMF solvent the LHE of them increases to different degrees. The higher LHE will lead to higher $J_{SC}$, therefore, LS-387 will have better photoelectric conversion performance due to its higher LHE.

In addition, the influence of the electron injection efficiency of the excited state ($\phi_{inject}$) on the $J_{SC}$ was evaluated. The $\phi_{inject}$ is closely related to the driving force of electron injection ($∆G_{inject}$). The Marcus theory determines the electron transfer ability of an excited state dye into a semiconductor [48,49]:

(9) $$\kappa_{inject}=\left[V_{RP}\right]\frac{2}{h}{\left(\frac{\pi }{\lambda k_{B}T}\right)}^{\frac{1}{2}}\exp\left[-\frac{(∆G_{inject}+\lambda ){}^{2}}{4\lambda k_{B}T}\right]$$

where $\kappa_{inject}$ is the rate constant (in $S^{-1}$) of the electron injection from dye to ${\mathrm{TiO}}_{2}$, h is the Planck constant, $k_{B}$ is the Boltzmann constant, $∆G_{inject}$ is the electron injection free energy, *λ* is the reorganization energy. |$V_{RP}$| is the coupling constant between the reagent and the product potential curves. It can be concluded from the above equation that a larger |$V_{RP}$| will increase $\kappa_{inject}$ and result in faster electron injection. Hsu et al. have given the equation for $\left|V_{RP}\right|$ [50]:

(10) $$\left|V_{RP}\right|=\frac{∆E_{RP}}{2}$$

According to Koopmans approximation, the $∆E_{RP}$ is derived from [51,52]:

(11) $$∆E_{RP}=E_{0-0}^{dye}-\left[2E_{ox}^{dye}+E_{RED}^{dye}+E_{CB}^{TiO_{2}}\right]$$

Preat’s theoretical method shows the calculation method of $∆G_{inject}$ [53]:

(12) $$∆G^{inject}=E_{ox}^{dye*}-E_{CB}^{SC}$$

Here $E_{ox}^{dye*}$ represents the oxidation potential of the dye in the excited state, $E_{CB}^{SC}$ represents the reduction potential of ${\mathrm{TiO}}_{2}$ semiconductor [54] ($E_{CB}^{SC}$ = 4.0 eV) Thereunto $E_{ox}^{dye*}$ can also be expressed as:

(13) $$E_{ox}^{dye*}=E_{ox}^{dye}-\lambda_{max}$$

where $E_{ox}^{dye}$ represents the oxidation reduction potential of the ground state, $\lambda_{max}$ represents the energy of the ICT. Higher oxidation potentials can result in greater driving force for the injection.

As show in Table 5, the $E_{ox}^{dye*}$, the $∆G_{inject}$, and |$V_{RP}$| of LS-385, LS-386 and LS-387 were calculated. The oxidation potential ($E_{ox}^{dye*}$) of the three dyes in vacuum is as follows: LS-387 (2.364 eV) < LS-386 (2.658 eV) ≈ LS-385 (2.654 eV), and in solvent is as follows: LS-387 (2.449 eV) < LS-386 (2.677 eV) < LS-385 (2.681 eV); so LS-387 has a lower value of $E_{ox}^{dye*}$ in vacuum and solvent. Lower $E_{ox}^{dye*}$ will result in easier photooxidation.

The value of $∆G^{inject}$ is negative, which means that dye excited states can easily inject electrons into ${\mathrm{TiO}}_{2}$CB. As shown in Table 5, the absolute value of the $∆G^{inject}$ of the three dyes in vacuum can be arranged in sequence: LS-387 (1.636 eV) > LS-385 (1.346 eV) > LS-386 (1.342 eV), and the $∆G^{inject}$ of the three molecules is higher than 0.2 eV, which also shows that the molecular excited states can smoothly inject electrons into the ${\mathrm{TiO}}_{2}$CB. Moreover, the absolute value of $∆G^{inject}$ for LS-387 is much larger than LS-385 and LS-386. Therefore, LS-387 has the higher $∆G^{inject}$; Table 5 also lists the coupling constant ($V_{RP}$) in vacuum and solvent, in which LS-387 has the higher $V_{RP}$ compared with LS-385 and LS-386. Therefore, LS-387 will produce a higher $J_{SC}$ and further improve efficiency.

The dye regeneration free energy ($∆G_{dye}^{regen}$) can be used to characterize the regeneration ability of dye molecules from $I^{-}$/$I^{3-}$ electrolyte; the higher the $∆G_{dye}^{regen}$ drive, the better the regenerative capacity and electron transport capacity of the dye become. $∆G_{dye}^{regen}$ can be calculated as the difference between the redox potential of $I^{-}$/$I^{3-}$ ($E_{redox}^{electrolyte}$ = −4.60 eV) [55,56] and $E_{ox}^{dye}$. Table 5 shows the $∆G_{dye}^{regen}$ of the three dyes can be arranged: LS−385 (0.995 eV) > LS−386 (0.964 eV) > LS−387 (0.525 eV); and dyes in the solvent follow the same sequence. The values of $∆G_{dye}^{regen}$ of the three dyes in vacuum and solvent are higher than 0.5 eV, which means that the three dyes can finalize the regenerative process.

An important parameter to study charge transfer efficiency is the excited state lifetime (τ), which can be evaluated via the following equation:

(14) $$\tau =\frac{1.499}{f\times E^{2}}$$

where *E* represents the excitation energy of the different electronic states (${\mathrm{cm}}^{-1}$) and f is the oscillator strength. Relevant data are in Table 6. The τ of the three dyes are arranged in sequence: LS 386 (1.82 ns) < LS-385 (2.02 ns) < LS-387 (2.29 ns). Intuitive data shows that LS-387 maintains long-term stability in the cationic state.

**Figure 6:**
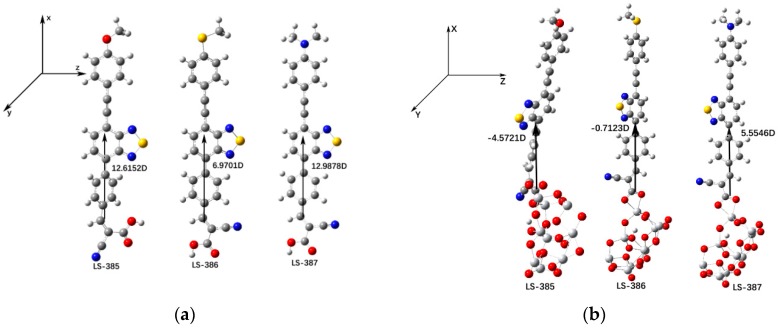
(**a**) Calculated vertical dipole moment $\mu_{normol}$ of dye in DMF solvent. (**b**) Calculated vertical dipole moment $\mu_{normol}$ of dye/${\left({\mathrm{TiO}}_{2}\right)}_{9}$ complexes in DMF solvent.

The $V_{OC}$ represents the difference between the quasi-Fermi level (electrons in the titanium dioxide conduction band) and the redox potential (electrolyte) [57]. Movement of the $E_{CB}$ after the dye adsorption on the semiconductor substrate directly affects the $V_{OC}$, and the relationship between the movement of the $E_{CB}$ and adsorbed molecular characteristics can be written as [31,58]:

(15) $$∆E_{CB}=\frac{-q\mu_{normol}\gamma }{\varepsilon_{0}\varepsilon }$$

where $\mu_{normol}$ is the dipole moment component of the dye molecules perpendicular to the surface of ${\mathrm{TiO}}_{2}$, γ is the absorption concentration of the semiconductor surface, and $\varepsilon_{0}$ and ε represent the dielectric constant and the organic monolayer in vacuum, respectively. The dyes with larger $\mu_{normol}$ and $∆E_{CB}$ will generate a larger $V_{OC}$. Figure 6a shows that for isolated dyes, the $\mu_{normol}$ (in Debye) of LS-387 (12.9878D) is the largest compared with LS-385 and LS-386; for dye/$\left({\mathrm{TiO}}_{2}\right)$_9_, the value of $\mu_{normol}$ should follow the sequence of LS-387 > LS-385 > LS-386 (see Figure 6b). Therefore, the high $V_{OC}$ of LS-387 can be contributed to the larger $\mu_{normol}$ of LS-387, which is in good agreement with the experimental results [16].

### 3.7. Analysis of Recombinant Active Site

**Figure 7:**
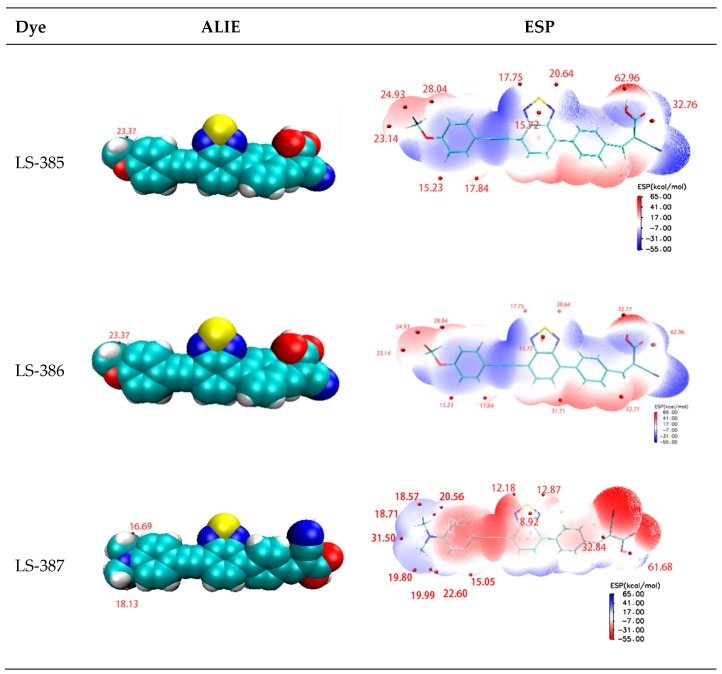
Graphical representation for minima of ALIE on VMD surface and local maxima of ESP on VMD surface of the dye cations of $\mathrm{LS}-385^{+}$, $\mathrm{LS}-386^{+}$, and $\mathrm{LS}-387^{+}$. The extrema ALIE (in eV) and ESP (in kcal/mol) points near to the donor regions are marked.

It is very important to gain electrons in the regeneration process of dyes. Molecular surface electrostatic potential (ESP) [59] and average local ionization energy (ALIE) [60] can be used to determine the active sites of electrolyte and dye cations in the active region. A higher ESP site means the site attracts the strongest nucleophilic agent and is most likely to interact with the negatively charged electrolyte [37]. One of the most important roles of ALIE is to predict the reaction sites for electrophilic or free radical attack. The minimum value of surface ALIE can effectively reveal which atoms are more likely to be the preferred location for electrophilic or free radical attack. The relevant data and images are summarized in Figure 7. As shown, the minima of ALIE distributed near the donor atom (O, S, N), and LS-387 has a smaller value than LS-385 and LS-386. Furthermore, in the donor site, the maxima ESP have a concentrated distribution near the N atom site of LS-387. It seems that the main atoms O, S, N of donor complement can serve as active sites for dye molecules and electrolytes. The extreme points of ALIE and ESP determine that the donor site may be a point of interest for electrolyte ions.

### 3.8. First Hyperpolarizability

The first static hyperpolarizability is viewed as third-order tensor and second-order nonlinear optical response (NLO) coefficient [61]. Table S3 shows that the direction of the first hyperpolarizability (positive value) is in the same direction as the X axis, which also means that the three molecules have the same direction of charge transfer. LS-387 has a higher first hyperpolarizability, which may be due to the planar structure between the π bridge and the acceptor (see Table 1). This facilitates the better ICT of electrons from the donor to the acceptor and accelerates electron injection from the dye molecules to the ${\mathrm{TiO}}_{2}$CB.

### 3.9. Molecular Design

**Figure 8:**
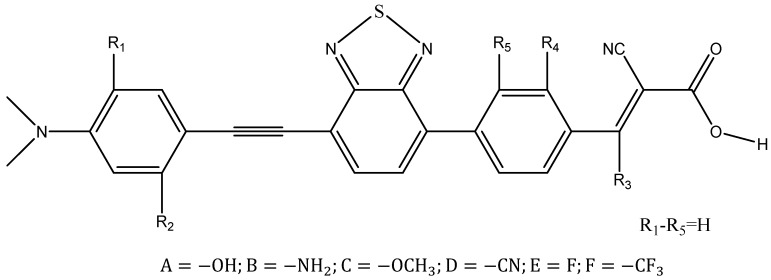
Molecular structures of dye LS-387 and designed molecules (LS-387-X and LS-387-Y).

By analyzing the photoelectric properties of the original dyes, we can obtain the conclusion that the parameters of LS-387 are superior to the others; as a result, LS-387 produces better PCE(*η* = 5.61% [16]). DFT provides a design strategy for controlling performance from the viewpoint of theory [60,61,62]. Based on LS-387, we theoretically designed fifteen new dye molecules to improve the electro-optical performance. On the donor group, we symmetrically introduced to the electron donating substituents (–OH, –NH_2_ and –OCH_3_); on the molecule’s acceptor group, the electron-acceptors (–CF_3_, –F and –CN). By introducing different groups, we reduced the molecular energy gap, which is conducive to a red-shift of the absorption spectrum; at the same time, the introduction of individual groups can improve the dye regeneration free energy to some extent, thus improving the regeneration efficiency ($\eta_{reg}$) and $J_{SC}$ of the dyes. On LS-387, we defined five positions ($R_{1}–R_{5}$) to introduce electron groups (see Figure 8). Also, in $R_{1}$ and $R_{2}$, three donor groups were introduced, where the molecules are named: LS-387-X (X = 1A, 1B, 1C, 12A, 12B and 12C); and in the acceptor group($R_{3}$, $R_{4}$, and $R_{5}$), we introduced three electron-acceptors, where the molecules are named: LS-387-Y (Y = 3D, 3E, 3F, 4D, 4E, 4F, 5D, 5E and 5F).

The molecules (LS-387-X and LS-387-Y) ground state were optimized in DMF solvent, and bond length and dihedral angle are listed in Tables S4 and S5. As shown in Table S4, the bond length ($d_{1}$ to $d_{6}$) of LS-387-1A (1B and 1C) is not obviously changed compared with LS-387; However, the $d_{1}$ of LS-387-12A (12B and 12C) is higher than LS-387. It seems that introducing into two of the same electron groups leads to larger bond length that can affect the stability of the molecules. In addition, in the acceptor group, the $d_{5}$ and $d_{6}$ of LS-387-3D (3E, 3F, 4D, 4E, 4F, 5D, 5E and 5F) is greater than LS-387, but the $d_{1}–d_{4}$ is not obviously changed compared with LS-387. Therefore, the electronic groups introduced by the acceptors are also not conducive to improving the stability of the molecules. As shown in Table S5, the dihedral angle (∠1) of LS-387-X (X = 1A, 1B, 1C, 12A, 12B and 12C) is not obviously changed compared to LS-387; for the dihedral angle ∠2, the LS-387-12C (−0.062) has a smaller value than LS-387, and the molecule is more planar in the acceptor site, which is beneficial to ICT. For LS-387-Y, due to the interatomic repulsive effect of the group, the increases of ∠1 and ∠2 will be different, and the ICT will have a negative effect.

**Figure 9:**
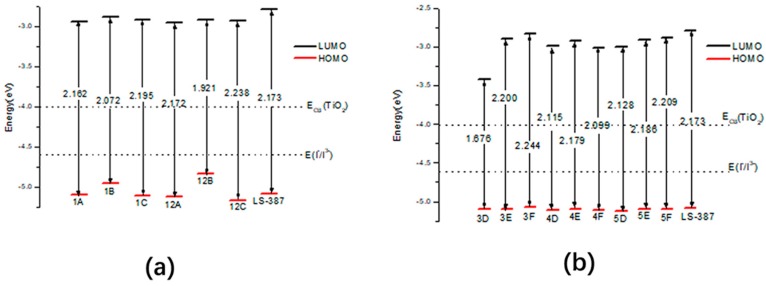
Energy level in solvent of LS-387-based designed molecules, where (**a**) is LS-387-X (X = 1A, 1B, 1C 12A, 12B and 12C), (**b**) is LS-387-Y (Y = 3D, 3E, 3F, 4D, 4E, 4F, 5D, 5E and 5F).

Figure 9 shows the HOMO, LUMO energy level and the energy gap (∆G = |H − L|), and data are listed in Table S6. The energy gap of LS-387-1B and LS-387-12B is 2.072 eV and 1.921 eV, and the higher HOMO energy level of LS-387-1B and LS-387-12B will result in a small gap (see Figure 9a). Because a narrow energy gap is favorable to red-shift absorption, the smaller energy gap for LS-387-1B and LS-387-12B by introducing $–{\mathrm{NH}}_{2}$ will lead to a larger absorption peak. For acceptor designed molecules LS-387-Y, the LS-387-3D (1.676 eV), LS-387-4D (2.115 eV), and LS-387-5D (2.128 eV) have a lower gap (see Figure 9b), which is due to lower LUMO level for LS-387-Y (3D, 4D and 5D). To sum up, the introduction of the $–{\mathrm{NH}}_{2}$ group at the donor site and the introduction of –CN at the acceptor site can reduce the gap, thus leading to a red-shift in the maximum absorption peak and improvement of the light trapping efficiency.

The excited state characteristics of the design molecules were calculated, and the results are listed in Table S7. As shown in Table S7, the LS-387-1B and the LS-387-12B have the $\lambda_{max}$ of 496.16 nm and 490.19 nm, which is larger than LS-387 (470.40 nm). So dye LS-387-1B and LS-387-12B have a red-shift of 20–25 nm. The first excited states of LS-387-1B and LS-387-12B show an electron transition of HOMO→LUMO (see Figure S2). While for the LS-387-3D, its $\lambda_{max}$ (an electron transition is HOMO→LUMO) is found to be 485.58 nm, which configuration will produce a larger red-shift relative to the original molecule. Figure S3 shows absorption spectra of 15 designed molecules, which show that LS-387-1B and LS-387-12B have obvious red-shifted absorption (LS-387-3D has an obvious absorption peak red-shifted relative to the original molecules). In summary, it was found that the design by introducing the $–{\mathrm{NH}}_{2}$ group individually or in pairs on the donor site should reduce the energy gap and make the spectrum red-shifted, and then improve the ICT; introducing on the acceptor site $R_{3}$ position –CN groups has a similar trend.

From Section 3.4, IP and EA are important injection parameters, and $E_{fund}$ can be used to characterize the electronic contribution of dye molecules [62,63,64]. The LS-387-1B (12A, 12B and 12C) has a lower value of IP compared with LS-387 (see Table S8), and the EA of LS-387-3D has a large value relative to LS-387 and other designed molecules. Therefore, LS-387-1B (12A, 12B and 12C) will produce a higher outcome of extracting electrons, and LS-387-3D will have a better absorbing ability of electrons. The $E_{fund}$ of LS-387-3D produced a lower value relative to the original molecule and other designed molecules, therefore, LS-387-3D will show better electronic ability.

On the basis of the ground state optimizations of fifteen designed molecules, four electrochemical activity parameters are also listed in Table S8. The h of LS-387-1B (12A, 12B and 12C) has a significant decrease compared with LS-387 (0.88). Among the above three designed dyes, for LS-387-1B (12B) the introduction of $–{\mathrm{NH}}_{2}$ in the donor terminal reduces the chemical hardness of the dyes more effectively than other introductions of $–{\mathrm{OCH}}_{3}$ and –OH. The LS-387-3D (4D and 5D) and the LS-387-3E also has a lower h compared with LS-387. In summary, introduction of two electron groups ($–{\mathrm{NH}}_{2}$) in the donor site or the introduction of $R_{3}$ ($–{\mathrm{CF}}_{3}$ and –CN) in the acceptor are more beneficial to reducing the h of the dye molecules. Moreover, the dyes of LS-387-12A (12B and 12C) have a higher $\omega^{+}$ compared to LS-387 and LS-387-1A (1B and 1C). LS-387-3D (13.17) and LS-387-3F (9.76) also has a maximum value of $\omega^{+}$. So, introduction of electron-donating groups ($–{\mathrm{NH}}_{2}$) in pairs on the donor site and introduction of $–{\mathrm{CF}}_{3}$ and ‒CN in the acceptor site $R_{3}$ were beneficial to increase the $\omega^{+}$ dye molecules. Therefore, the above two parameters indicated that LS-387-12B and LS-387-3D (3F) would have a higher $J_{SC}$. The higher absolute value of $∆G^{inject}$ can lead spontaneously to inject electrons to TiO_2_. Table 7 shows that LS-387-1B (12B) has a higher absolute value of $∆G^{inject}$ compared with dye LS-387. The higher HOMO levels of LS-387-1B (12B) lead to greater $∆G^{inject}$ (see Figure 9a). The introduction of the $–{\mathrm{NH}}_{2}$ group is helpful to increase the electron injection, and introduction of two $–{\mathrm{NH}}_{2}$ groups can still further increase the electron injection compared with single introduction of $–{\mathrm{NH}}_{2}$. Also, the coupling constant ($V_{RP}$) is listed in Table 7. For serials LS-387-X, LS-387-1B (12B) has the higher $V_{RP}$ compared with LS-387 and the other five dyes, which means that faster electron injection can occur for LS-387-1B (12B). The dye regeneration free energy ($∆G_{dye}^{regen}$) has important effects on the PCE. As shown in Table 7, for LS-387-X, LS-387-12A (12C) has a higher value of $∆G_{dye}^{regen}$ compared with other dyes, indicating that two groups –OH and $–{\mathrm{OCH}}_{3}$ are beneficial to improve the free energy of the dye regeneration. In addition, for LS-387-3D (4D and 5D), the $∆G_{dye}^{regen}$ is large than LS-387, showing that the –CN radical group is helpful to improve the free energy of the dye regeneration. The larger $∆G_{dye}^{regen}$ is beneficial to improve $\eta_{reg}$ and increase $J_{SC}$. Considering the above three properties ($∆G^{inject}$, $∆G_{dye}^{regen}$ and LHE), the $J_{SC}$ of LS-387-1B (12B) and LS-387-3D (4D and 5D) will be better than LS-387.

Table 7 shows the $\mu_{normol}$ of fifteen designed molecules, and for LS-387-X, the LS-387-1B has a higher $\mu_{normol}$ compared with LS-387; for LS-387-Y, the dyes of LS-387-4D (4E and 4F) have higher $\mu_{normol}$ compared with LS-387. In summary, introduction of electron-donating groups ($–{\mathrm{NH}}_{2}$) on the donor site and introduction of electron-acceptor groups (–CN, –F and $–{\mathrm{CF}}_{3}$) on the acceptor site $R_{4}$ are beneficial for the improvement of $\mu_{normol}$, which then improves the $V_{OC}$.

## 4. Conclusions

This paper systematically studied the photoelectric properties of three basic dye molecules with DFT calculations from the following aspects (structures, energy levels, spectra, electron transfer process, and chemical parameters etc.). We inferred some common conclusions: (a) LS-387 has a shorter bond length and smaller dihedral angle, which is of benefit to the ICT. (b) LS-387 displays higher HOMO and narrow gap, which leads to stronger electron donating ability and a broad absorption spectrum; (c) Analysis by CDD shows that LS-387 can effectively improve charge separation, which is supported by NBO analysis; (d) the well experimental performance of $J_{SC}$ and $V_{OC}$ for LS-387 can contribute to higher $∆G^{inject}$ and LHE as well as $\mu_{normol}$; (e) fifteen dyes were designed with pull-push electron groups in donor and acceptor, and it was found that the rational introduction of electron groups ($–{\mathrm{NH}}_{2},\;–\mathrm{CN}$, –F and $–{\mathrm{CF}}_{3}$) in the molecule can effectively improve the photoelectric properties of dyes. The above discussion can provide a theoretical basis for achieving higher performance of DSSC.

## Figures and Tables

**Table 1:** bond lengths (Å) $d_{1}$ to $d_{6}$ and dihedral angles (°) of dyes in vacuum and DMF solvent, respectively.

	LS-385		LS-386		LS-387	
	Vacuum	Solvent	Vacuum	Solvent	Vacuum	Solvent
$d_{1}$(Å)	1.360	1.358	1.777	1.777	1.379	1.371
$d_{2}$(Å)	1.420	1.421	1.419	1.421	1.416	1.416
$d_{3}$(Å)	1.413	1.415	1.413	1.415	1.410	1.411
$d_{4}$(Å)	1.477	1.477	1.477	1.477	1.476	1.476
$d_{5}$(Å)	1.458	1.455	1.452	1.450	1.451	1.448
$d_{6}$(Å)	1.494	1.492	1.489	1.488	1.488	1.488
∠1	32.4	34.4	33.2	34.6	32.4	33.5
∠2	26.8	25.5	1.4	0.7	1.5	0.5

**Table 2:** The excitation energy and oscillator strength obtained by TD-DFT//Cam-B3LYP/6–31G(d) in vacuum and N,N-dimethylformamide (DMF) solvent.

	Dye	State	$E_{g}$ $/\lambda_{max}$	F	Main CI
Gas	LS-385	$S_{1}$	2.9410/421.57	1.1646	0.65655(H→L)
		$S_{2}$	3.8832/319.29	0.5094	0.54620(H→L + 1)
		$S_{3}$	4.1821/296.46	0.0214	0.57628(H-1→L)
	LS-386	$S_{1}$	2.9060/426.65	0.7495	0.66540(H→L)
		$S_{2}$	3.8161/324.90	0.0063	0.43530(H-9→L)
		$S_{3}$	4.0477/306.31	0.7171	0.51222(H→L + 1)
	LS-387	$S_{1}$	2.7606/449.11	1.2886	0.62767(H→L)
		$S_{2}$	3.7304/332.36	0.4445	0.55969(H→L + 1)
		$S_{3}$	3.9155/316.65	0.0101	0.58567(H-1→L)
Solvent	LS-385	$S_{1}$	2.8936/428.47	1.3613	0.64539(H→L)
		$S_{2}$	3.7665/329.18	0.4518	0.52513(H→L + 1)
		$S_{3}$	4.1529/298.55	0.0581	0.53107(H-1→L)
	LS-386	$S_{1}$	2.8912/428.83	1.5163	0.62471(H→L)
		$S_{2}$	3.7748/328.46	0.4001	0.50349(H→L + 1)
		$S_{3}$	4.0716/304.51	0.1023	0.48781(H-1→L)
	LS-387	$S_{1}$	2.6357/470.40	1.4452	0.61544(H→L)
		$S_{2}$	3.5912/345.25	0.4431	0.52970(H→L + 1)
		$S_{3}$	3.7958/326.63	0.0016	0.53890(H-1→L)
Solvent + ${\mathrm{TiO}}_{2}$	LS-385	$S_{1}$	2.8183/439.92	1.5757	0.44929(H→L + 1)
		$S_{2}$	3.5424/350.00	0.2339	0.31156(H→L + 7)
		$S_{3}$	4.0393/306.94	0.0108	0.37487(H→L)
	LS-386	$S_{1}$	2.8292/438.23	1.8788	0.54394(H→L)
		$S_{2}$	3.6151/342.96	0.3837	0.28510(H→L + 7)
		$S_{3}$	4.0090/309.26	0.0084	0.41495(H-1→L)
	LS-387	$S_{1}$	2.5615/484.02	1.7597	0.50407(H→L)
		$S_{2}$	3.4105/363.53	0.3750	0.30764(H→L + 8)
		$S_{3}$	3.6944/335.60	0.0863	0.43001(H-1→L)

**Table 3:** Natural Bond Orbital Analysis (Atomic Charge in a.u.) for the Ground State ($S_{0}$) and Excited State ($S_{1}$) of the dyes with D-π-A-π-A fragments.

Dye		Donor	π	Acetylene Bridge	BTZ	Acceptor
LS-385	$S_{0}$	−0.18	0.30	0.02	−0.07	−0.06
	$S_{1}$	−0.16	0.46	0.11	−0.27	−0.14
	∆q	−0.02	−0.16	−0.09	0.20	0.08
LS-386	$S_{0}$	0.14	−0.04	0.03	−0.05	−0.08
	$S_{1}$	0.28	0.004	0.11	−0.23	−0.16
	∆q	−0.14	-0.036	−0.08	0.18	0.08
LS-387	$S_{0}$	0.02	0.14	0.004	−0.07	−0.09
	$S_{1}$	0.13	0.31	0.09	−0.36	−0.18
	∆q	−0.11	−0.17	−0.086	0.29	0.09

**Table 4:** The reorganization energy ($\lambda_{h}$ and $\lambda_{e}$) and the chemical reactivity parameters $\lambda_{h}$ and $\lambda_{e}$ of LS-385, LS-386 and LS-387 in vacuum and DMF solvent.

	LS-385		LS-386		LS-387	
	Vacuum	Solvent	Vacuum	Solvent	Vacuum	Solvent
$\lambda_{h}$	0.25	0.24	0.20	0.19	0.18	0.13
$\lambda_{e}$	0.37	0.34	0.42	0.29	0.33	0.27
h	2.37	1.09	2.32	1.08	2.22	0.88
*ω*	3.91	8.41	4.01	8.57	3.64	9.27
$\omega^{+}$	2.05	6.41	2.14	6.56	1.91	7.36
$\omega^{-}$	6.35	10.69	6.46	10.86	5.93	11.40

**Table 5:** The $V_{RP}$, the light harvesting efficiencies (LHE), the electron injection free energy ($∆G^{inject}$, in eV), the oxidation potential of the dye in ground state ($E_{ox}^{dye*}$, in eV), the oxidation potential of the dye in excited state ($E_{ox}^{dye}$, in eV), the dye regeneration free energy ($∆G_{dye}^{regen}$, in eV), and the vertical dipole moment of ($\mu_{normol}$, in Debye) in vacuum and DMF solvent.

	LS-385		LS-386		LS-387	
	Vacuum	Solvent	Vacuum	Solvent	Vacuum	Solvent
$V_{RP}$	0.673	0.660	0.671	0.662	0.818	0.776
LHE	0.9315	0.9565	0.8220	0.9695	0.9485	0.9641
$∆G^{inject}$	−1.346	−1.319	−1.342	−1.324	−1.636	−1.551
$∆G_{dye}^{regen}$	0.995	0.975	0.964	0.968	0.525	0.485
$E_{ox}^{dye}$	5.595	5.575	5.564	5.568	5.125	5.085
$E_{ox}^{dye*}$	2.654	2.681	2.658	2.677	2.364	2.449
$\mu_{normol}$	−10.6528	−12.6125	−6.1017	−6.7901	−10.5313	−12.9878

**Table 6:** The excited state lifetime of (τ, in ns) in DMF solvent.

	LS-385	LS-386	LS-387
τ (ns)	2.02	1.82	2.29

**Table 7:** Chemical parameters in DMF solvent. ($∆G^{inject}$, $∆G_{dye}^{regen}$, $E_{ox}^{dye}$ and $E_{ox}^{dye*}$, in eV; $\mu_{normol}$, in Debye).

Dyes	LHE	$∆G^{inject}$	$∆G_{dye}^{regen}$	$E_{ox}^{dye}$	$E_{ox}^{dye*}$	$V_{RP}$	$\mu_{normol}$
LS-387-1A	0.9636	−1.512	0.499	5.099	2.489	0.756	11.5551
LS-387-1B	0.9585	−1.538	0.361	4.961	2.462	0.769	13.3062
LS-387-1C	0.9634	−1.529	0.509	5.109	2.471	0.765	12.9133
LS-387-3D	0.9584	−1.453	0.500	5.100	2.547	0.727	12.5191
LS-387-3E	0.9563	−1.558	0.493	5.093	2.442	0.779	12.9292
LS-387-3F	0.9349	−1.650	0.470	5.070	2.350	0.825	9.1245
LS-387-4D	0.9503	−1.538	0.508	5.108	2.462	0.769	14.0796
LS-387-4E	0.9605	−1.531	0.499	5.099	2.469	0.766	13.8853
LS-387-4F	0.9598	−1.510	0.505	5.105	2.490	0.755	13.1415
LS-387-5D	0.9458	−1.574	0.525	5.125	2.426	0.787	10.6711
LS-387-5E	0.9500	−1.628	0.498	5.098	2.372	0.814	11.4385
LS-387-5F	0.9272	−1.713	0.495	5.095	2.287	0.857	11.0183
LS-387-12A	0.9609	−1.545	0.524	5.124	2.455	0.773	7.3461
LS-387-12B	0.9323	−1.687	0.242	4.842	2.313	0.844	10.3030
LS-387-12C	0.9642	−1.503	0.566	5.166	2.497	0.752	12.3172

## Supplementary Materials

The following are available online at http://www.mdpi.com/1996-1944/11/10/2027/s1, Figure S1. Charge difference density (CDD) of the selected excited state for dyes in solvent. (Green and red stand for the hole and electron, respectively), Figure S2. Frontier molecular orbital of molecular designing in DMF solvent. Figure S3. The UV-Vis absorption spectrum in DMF solvent. Table S1. Energy levels of HOMO and LUMO and energy gaps calculated by DFT in vacuum and DMF solvent of three dyes. Table S2. The ionization potentials(IP) and electron affinities(EA) of LS-385, LS-386 and LS-387 in vacuum and solvent. Table S3. Calculated the static first hyperpolarizability of the three dyes in vacuum and DMF solvent. Table S4. The bond length of LS-387 analogous in DMF solvent. Table S5. Dihedral angle in DMF solvent. Table S6. The energy level and the energy gap in DMF solvent. Table S7. Transition energies ($E_{g}$) and oscillator strengths of 15 designed molecules in DMF solvent. Table S8. The Electrochemical Parameter in DMF solvent. Cartesian coordinates of the optimized structure.
