# Supplementary material for: Photoactuated Properties of Acetylene-Congeners Non-Metallic Dyes and Molecular Design for Solar Cells
Source: Materials (Basel). 2018 Oct 18;11(10):2027. doi: 10.3390/ma11102027 (PMC6213635; doi:10.3390/ma11102027)
Supplement: Supplementary file 1 [file materials-11-02027-s001.pdf]

Supporting Information

Figure S1. Charge difference density（CDD） of the selected excited state for dyes in solvent. (Green and red stand for the hole and electron, respectively)

	LS-385	LS-386	LS-387
S_1	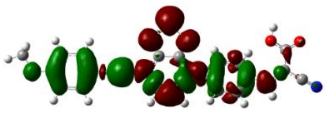	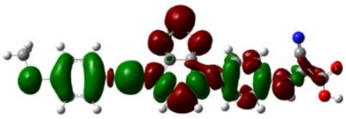	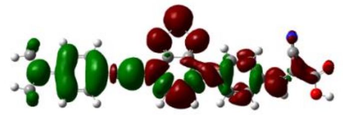
S_2	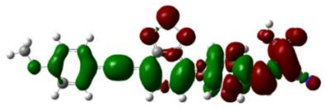	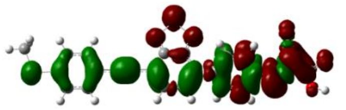	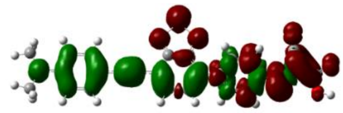
S_3	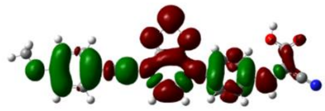	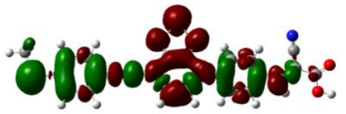	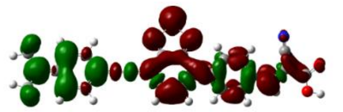
S_4	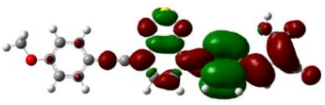	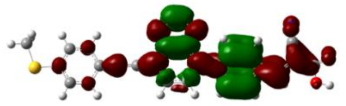	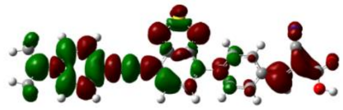
S_5	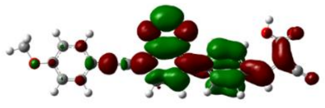	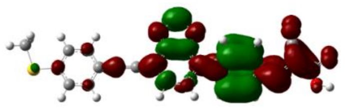	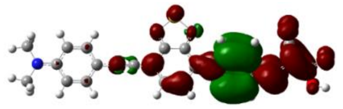
S_6	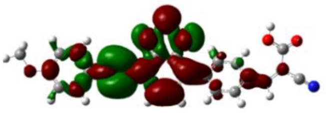	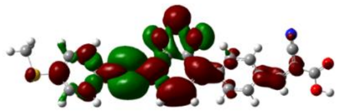	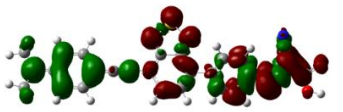

Figure S2. Frontier molecular orbital of molecular designing in DMF solvent.

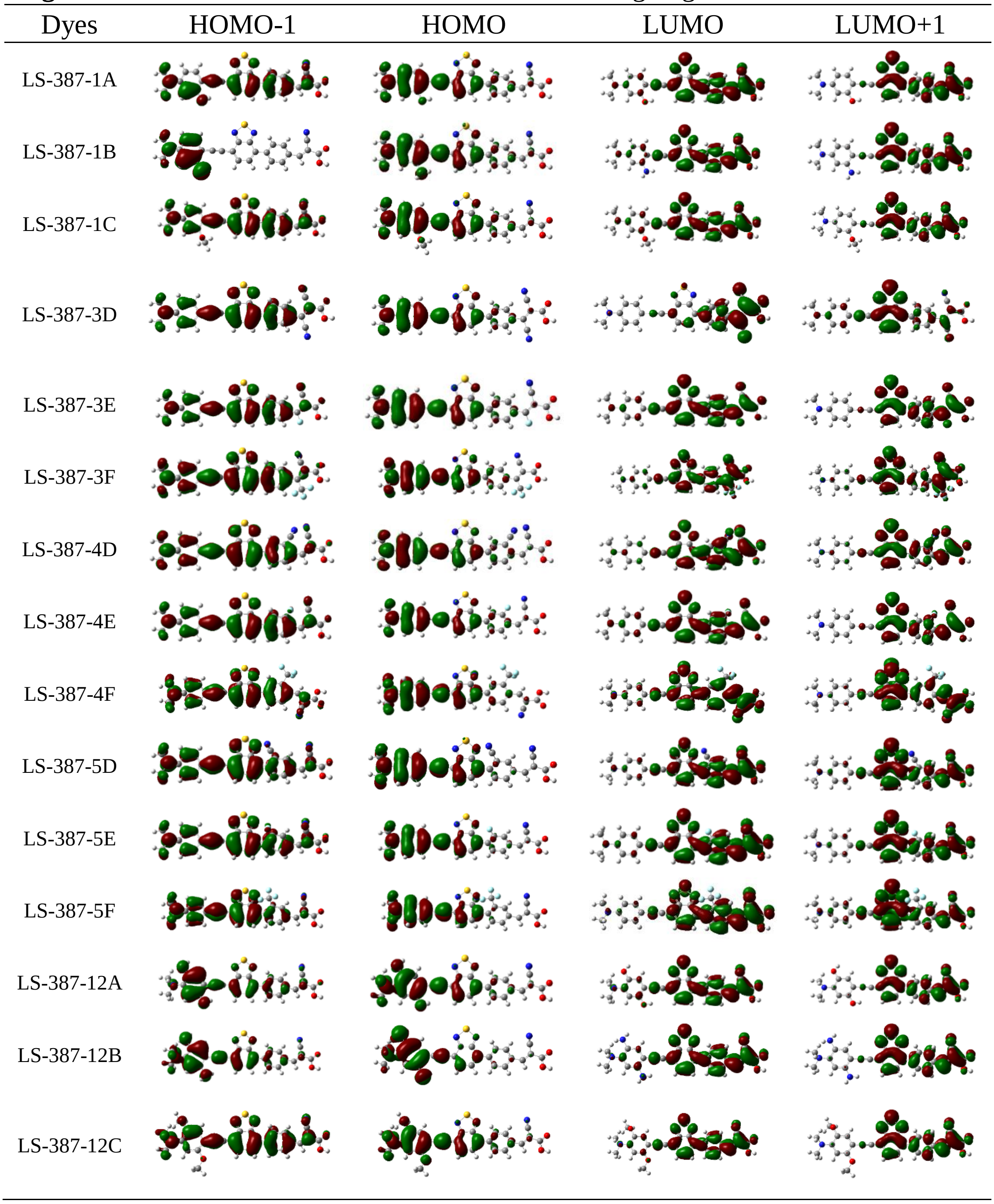

Figure S3. The UV-Vis absorption spectrum in DMF solvent.

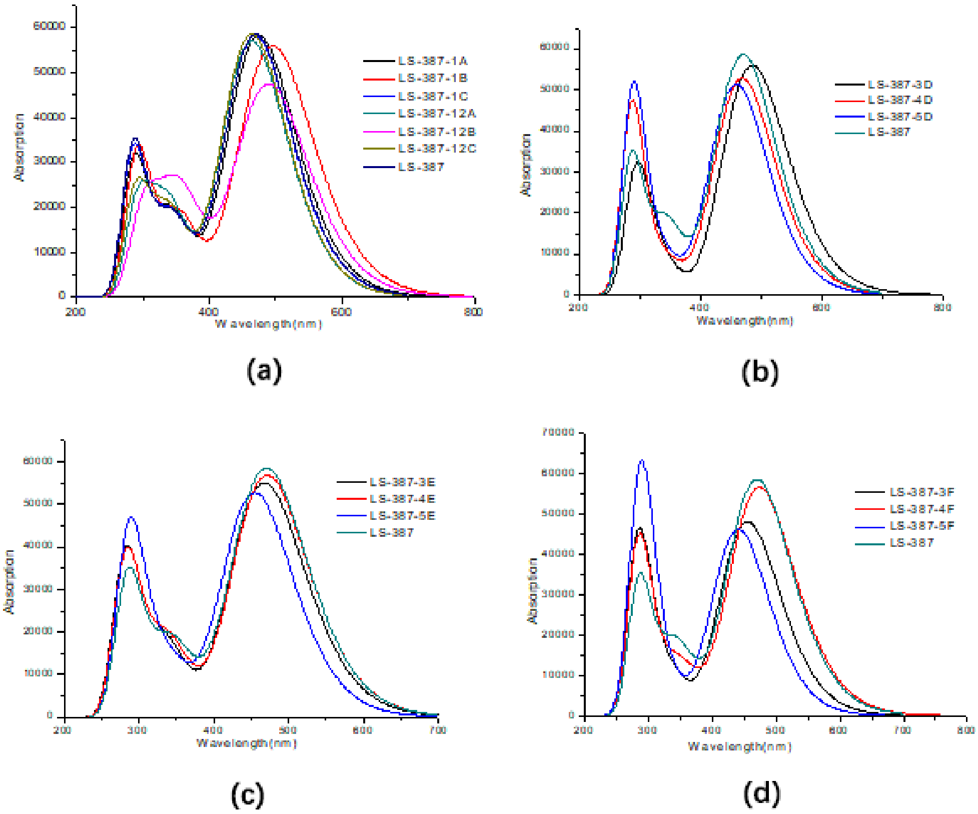

Table S1. Energy levels of HOMO and LUMO and energy gaps calculated by DFT in vacuum and DMF solvent of three dyes.

	LS-385			LS-386			LS-387		
	Gas	Solvent	Solvent+TiO_2	Gas	Solvent	Solvent+TiO_2	Gas	Solvent	Solvent+TiO_2
LUMO	-2.918	-2.909	-3.306	-2.962	-2.974	-3.290	-2.789	-2.912	-3.286
HOMO	-5.595	-5.574	-5.592	-5.564	-5.568	-5.581	-5.125	-5.085	-5.100
Gap	2.677	2.666	2.287	2.602	2.593	2.291	2.336	2.173	1.814

Table S2. The ionization potentials(IP) and electron affinities(EA) of LS-385、LS-386 and LS-387 in vacuum and solvent.

	LS-385		LS-386		LS-387	
	Vacuum	Solvent	Vacuum	Solvent	Vacuum	Solvent
IP	6.67	5.37	6.64	5.38	6.23	4.92
EA	1.93	3.19	1.99	3.22	1.80	3.16
E_{fund}	4.74	2.18	4.65	2.16	4.43	1.76

Table S3. Calculated the static first hyperpolarizability of the three dyes in vacuum and DMF solvent.

Condition	Dye	β_{xxx}	β_{xxy}	β_{xyy}	β_{xxz}	β_{xxz}	β_{xyz}	β_{yyz}	β_{xzz}	β_{yzz}	β_{zzz}	β_{tot}[a]
vacuum	LS-385	26282.2	-203.891	-135.538	40.2345	-31.7304	-29.9303	37.5371	-28.241	-40.6823	-5.84997	261.8738
	LS-386	-20227.9	-448.106	102.749	159.257	-103.914	8.73563	-7.70606	51.8503	8.9207	-10.6101	306.0448
	LS-387	-61925.5	-1270.77	280.604	132.478	342.417	101.456	-4.30704	106.019	2.57344	1.57896	1282.47
solvent	LS-385	51376	189.282	-330.65	114.838	-147.35	-70.277	72.397	-49.889	-84.347	-18.826	50996.02
	LS-386	-37325	-661.17	265.531	338.28	-334.06	26.9083	-24.764	91.2125	13.1034	-6.69	36971.56
	LS-387	-153237	-2625	741.533	243.375	704.728	250.918	-25.603	211.178	-6.6072	2.2261	152304.5

a: Calculation formula($\beta_{tot} = \sqrt{(\beta_{xxx} + \beta_{xyy} + \beta_{xzz})^2 + (\beta_{yyy} + \beta_{xxy} + \beta_{yzz})^2 + (\beta_{zzz} + \beta_{xxz} + \beta_{yyz})^2}$).

Table S4. The bond length of LS-387 analogous in DMF solvent.

Dyes	d_1(Å)	d_2(Å)	d_3(Å)	d_4(Å)	d_5(Å)	d_6(Å)
LS-387-1A	1.370	1.411	1.411	1.475	1.445	1.488
LS-387-1B	1.372	1.409	1.409	1.475	1.448	1.487
LS-387-1C	1.370	1.414	1.411	1.476	1.449	1.488
LS-387-3D	1.371	1.415	1.411	1.475	1.474	1.497
LS-387-3E	1.371	1.416	1.411	1.476	1.457	1.490
LS-387-3F	1.372	1.416	1.412	1.478	1.478	1.517
LS-387-4D	1.371	1.415	1.411	1.476	1.462	1.494
LS-387-4E	1.371	1.415	1.411	1.475	1.450	1.491
LS-387-4F	1.371	1.415	1.411	1.475	1.458	1.491
LS-387-5D	1.371	1.415	1.411	1.478	1.454	1.491
LS-387-5E	1.372	1.416	1.412	1.477	1.452	1.490
LS-387-5F	1.372	1.417	1.413	1.487	1.454	1.491
LS-387-12A	1.399	1.413	1.412	1.476	1.449	1.488
LS-387-12B	1.416	1.414	1.411	1.475	1.448	1.488
LS-387-12C	1.397	1.414	1.412	1.476	1.449	1.488

Table S5. Dihedral angle in DMF solvent.

Dye	∠C1	∠C2
LS-387-1A	-146.7	0.3
LS-387-1B	-147.3	0.4
LS-387-1C	-146.3	0.7
LS-387-3D	-146.4	-35.8
LS-387-3E	-146.0	-26.3
LS-387-3F	-144.8	-46.5
LS-387-4D	148.3	44.3
LS-387-4E	-149.5	-28.8
LS-387-4F	148.2	155.9
LS-387-5D	-127.1	-1.4
LS-387-5E	-130.4	0.2
LS-387-5F	-116.9	-3.9
LS-387-12A	-146.1	0.5
LS-387-12B	-146.6	0.4
LS-387-12C	-146.7	-0.1

Table S6. The energy level and the energy gap in DMF solvent.

Dye	LUMO	HOMO	Gap
LS-387-1A	-2.937	-5.099	2.162
LS-387-1B	-2.888	-4.961	2.072
LS-387-1C	-2.914	-5.109	2.195
LS-387-3D	-3.424	-5.100	1.676
LS-387-3E	-2.893	-5.093	2.200
LS-387-3F	-2.826	-5.070	2.244
LS-387-4D	-2.993	-5.108	2.115
LS-387-4E	-2.920	-5.099	2.179
LS-387-4F	-3.007	-5.105	2.099
LS-387-5D	-2.997	-5.125	2.128
LS-387-5E	-2.912	-5.098	2.186
LS-387-5F	-2.886	-5.095	2.209
LS-387-12A	-2.952	-5.124	2.172
LS-387-12B	-2.921	-4.842	1.921
LS-387-12C	-2.928	-5.166	2.238

Table S7. Transition energies (E_g) and oscillator strengths of 15 designed molecules in DMF solvent.

Dye	State	E_g/λ_{max}	f	Main configurations
LS-387-1A	S_1	2.6105/474.94	1.4389	0.62223(H→L)
	S_2	3.5846/345.88	0.4583	0.53791(H→L+1)
	S_3	3.8061/325.75	0.0023	0.52099(H-1→L)
LS-387-1B	S_1	2.4989/496.16	1.3818	0.62570(H→L)
	S_2	3.4886/355.39	0.4346	0.56798(H→L+1)
	S_3	3.7415/331.37	0.0269	0.58147(H-2→L)
LS-387-1C	S_1	2.6382/469.95	1.4365	0.61726(H→L)
	S_2	3.5978/344.61	0.4507	0.52988(H→L+1)
	S_3	3.8113/325.30	0.0019	0.53702(H-1→L)
LS-387-3D	S_1	2.5533/485.58	1.3811	0.52110(H→L)
	S_2	3.1467/394.01	0.0008	0.53026(H→L+1)
	S_3	3.5959/344.80	0.1494	0.50766(H-1→L)
LS-387-3E	S_1	2.6509/467.71	1.3594	0.62353(H→L)
	S_2	3.6746/337.41	0.4252	0.54355(H→L+1)
	S_3	3.8422/322.69	0.0001	0.55945(H-1→L)
LS-387-3F	S_1	2.7198/455.85	1.1862	0.60809(H→L)
	S_2	3.6897/336.03	0.2441	0.53389(H→L+1)
	S_3	3.9058/317.44	0.0081	0.55974(H-1→L)
LS-387-4D	S_1	2.6457/468.63	1.3040	0.59923(H→L)
	S_2	3.5838/345.96	0.2007	0.55859(H→L+1)

	S_3	3.8500/322.04	0.0104	0.56609(H-1→L)
LS-387-4E	S_1	2.6299/471.44	1.4038	0.62299(H→L)
	S_2	3.6423/340.40	0.4457	0.52144(H→L+1)
	S_3	3.8155/324.95	0.0017	0.53780(H-1→L)
LS-387-4F	S_1	2.6154/474.05	1.3961	0.60405(H→L)
	S_2	3.5456/349.68	0.3084	0.55860(H→L+1)
	S_3	3.7971/326.52	0.0365	0.56225(H-1→L)
LS-387-5D	S_1	2.6986/459.44	1.2657	0.58370(H→L)
	S_2	3.5835/345.99	0.1906	0.55890(H→L+1)
	S_3	3.9260/315.80	0.0863	0.55883(H-1→L)
LS-387-5E	S_1	2.7264/454.76	1.3012	0.58397(H→L)
	S_2	3.6186/342.63	0.2986	0.54672(H→L+1)
	S_3	3.8974/318.12	0.0585	0.54062(H-1→L)
LS-387-5F	S_1	2.8076/441.60	1.1379	0.53973(H→L)
	S_2	3.6338/341.19	0.1590	0.52888(H→L+1)
	S_3	4.0309/307.58	0.0868	0.51220(H-1→L)
LS-387-12A	S_1	2.6693/464.48	1.4073	0.61462(H→L)
	S_2	3.6244/342.08	0.4303	0.44842(H-1→L)
	S_3	3.7431/331.23	0.0666	0.38789(H→L+1)
LS-387-12B	S_1	2.5293/490.19	1.1694	0.61054(H→L)
	S_2	3.3835/366.44	0.3572	0.56864(H-1→L)
	S_3	3.5747/346.83	0.2565	0.52553(H→L+1)

	S_1	2.6690/464.53	1.4457	0.61716(H→L)
LS-387-12C	S_2	3.6270/341.84	0.4503	0.50549(H→L+1)
	S_3	3.8064/325.72	0.0159	0.49990(H-1→L)

Table S8. The Electrochemical Parameter in DMF solvent.

Dye	IP	EA	E_{fund}	h	ω	ω^{+}	ω^{-}
LS-387-1A	4.92	3.17	1.75	0.88	9.33	7.42	11.46
LS-387-1B	4.75	3.14	1.61	0.81	9.64	7.77	11.71
LS-387-1C	4.93	3.16	1.77	0.88	9.26	7.35	11.39
LS-387-3D	4.93	3.71	1.22	0.61	15.25	13.17	17.49
LS-387-3E	4.92	3.18	1.74	0.87	9.41	7.49	11.55
LS-387-3F	4.90	3.43	1.47	0.74	11.75	9.76	13.93
LS-387-4D	4.94	3.32	1.62	0.81	10.51	8.55	12.68
LS-387-4E	4.93	3.19	1.74	0.87	9.51	7.59	11.65
LS-387-4F	4.94	2.94	2.00	1.00	7.79	5.95	9.89
LS-387-5D	4.96	3.25	1.71	0.85	9.86	7.91	12.02
LS-387-5E	4.93	3.16	1.77	0.89	9.20	7.29	11.34
LS-387-5F	4.93	3.15	1.78	0.89	9.14	7.23	11.27
LS-387-12A	4.78	3.20	1.58	0.79	10.11	8.21	12.20
LS-387-12B	4.43	3.17	1.26	0.63	11.48	9.66	13.46
LS-387-12C	4.62	3.06	1.56	0.78	9.45	7.63	11.47

Cartesian coordinates of the optimized structure:

LS-385

	X	Y	Z
C	-5.99572600	-0.49174300	-0.04388900
C	-6.67276500	0.74065700	0.01036300
C	-8.06368500	0.80019000	0.00323000
C	-8.81031000	-0.38507400	-0.05900800
C	-8.14775200	-1.62344600	-0.11319100
C	-6.76494900	-1.67596000	-0.10582400
O	-10.16874100	-0.44445700	-0.07195300
C	-10.90200900	0.77180300	-0.02352900
C	-4.57694900	-0.53867600	-0.03622400
C	-3.35928900	-0.56174800	-0.02852700
C	-1.94706800	-0.58843900	-0.01906300
C	-1.21582500	-1.76615900	-0.07598500
C	0.20044500	-1.78509300	-0.06785900
C	0.98989000	-0.64758200	-0.00361600
C	0.27443600	0.60160700	0.05014500
C	-1.17992100	0.62964700	0.04862600
C	2.46444400	-0.73189400	0.01502800
C	3.10508200	-1.83018900	0.62095600
C	4.48636700	-1.95057000	0.60413300
C	5.30194200	-0.95947600	0.01955300
C	4.66043000	0.13333800	-0.59515100
C	3.27754400	0.24346700	-0.59662600
C	6.74131800	-1.19351800	0.02978600
N	-1.69730100	1.85753600	0.12103400
N	0.81939600	1.82199500	0.12857300
S	-0.42522500	2.89416600	0.18766100
C	7.82781600	-0.37143400	-0.05332400
C	9.11550000	-1.00138300	-0.14339600
N	10.13983500	-1.54910800	-0.20701700
C	7.90483600	1.12008600	-0.03508700
O	8.86248400	1.74442900	-0.43682800
O	6.82999300	1.71529700	0.53177300
H	-6.09454200	1.65806500	0.05866700
H	-8.55296300	1.76627400	0.04648900
H	-8.74557000	-2.52801800	-0.16085900
H	-6.25864300	-2.63528400	-0.14821500
H	-11.95465000	0.48549200	-0.04738500
H	-10.67897300	1.40978600	-0.88827100
H	-10.69746800	1.32787900	0.90035400
H	-1.74964800	-2.70893900	-0.13809200
H	0.68650500	-2.75286100	-0.14464400
H	2.51370900	-2.58773800	1.12578700
H	4.95233200	-2.81323000	1.07398900
H	5.24736300	0.89128200	-1.09713000
H	2.81499300	1.09193500	-1.08501200
H	6.99786900	-2.24985900	0.10188500
H	7.01706600	2.67298300	0.50868000

LS-386

S	0.00000000	0.00000000	0.00000000
C	0.00000000	0.00000000	1.81997139
C	1.21379773	0.00000000	2.51305581
C	1.21853045	-0.00133509	3.90944275
C	0.00779854	-0.00575610	4.61360639
C	-1.20627642	-0.00185207	3.91349851
C	-1.21083440	0.00349155	2.51843565
C	-0.01531400	-0.00701647	6.04711394
C	-0.08187985	0.00026960	7.27376506
C	-0.21563666	0.02446641	8.69871514
C	0.86595153	-0.07508566	9.52469366
C	0.72127550	-0.03791749	10.96228229
C	-0.48968603	0.11186523	11.58019833
C	-1.67208379	0.23043935	10.73429462
C	-1.53427712	0.17776906	9.29018987
C	-0.58680707	0.15682093	13.05968081
C	0.37347381	0.83909250	13.82170363
C	0.29262602	0.87740138	15.21243071
C	-0.76993411	0.26674857	15.90002972
C	-1.70036489	-0.45769259	15.14053461
C	-1.61212952	-0.50641148	13.74764923
C	-0.77066894	0.31645403	17.37018189
N	-2.63391951	0.28788637	8.60000168
N	-2.89073568	0.40900216	11.16220680
S	-4.08993421	0.49848412	9.73937420
C	-0.50827421	-1.73154061	-0.19683955
O	-0.42579131	0.47070415	20.24829139
C	-1.63822019	0.33834739	19.66848401
C	-1.85009873	0.25423789	18.19506202
O	-2.62282521	0.28182887	20.39448727
C	-3.20804649	0.15955529	17.71786471
N	-4.31290064	0.09031422	17.31671836
H	2.17115574	0.00167991	1.96722564
H	2.17677457	0.00128223	4.45386841
H	-2.16199003	-0.00151421	4.46275222
H	-2.17196871	0.00908625	1.97960788
H	1.87476868	-0.18639034	9.09358851
H	1.63909899	-0.15160609	11.56212870
H	1.20643305	1.36939929	13.33255110
H	1.07558068	1.41789518	15.76994819
H	-2.48945739	-1.04962042	15.62788816
H	-2.34487139	-1.12013007	13.19936953
H	0.21996397	0.44949072	17.83848634
H	-0.54078499	-1.97940588	-1.28126374
H	-1.52127239	-1.89284259	0.23449198
H	0.22071451	-2.40651553	0.30476386
H	-0.55534972	0.50113147	21.21063316

LS-387

N	-9.89095200	-0.27137600	0.15526500

C	-8.51270300	-0.31260900	0.12897500
C	-7.80786000	-1.52921900	0.31091900
C	-6.42386000	-1.56596500	0.27583200
C	-5.66142800	-0.39892300	0.06203100
C	-6.36114300	0.81345000	-0.11552800
C	-7.74451000	0.86025900	-0.08285200
C	-4.24618700	-0.43381600	0.02939400
C	-3.02731200	-0.44043800	-0.00143800
C	-1.61751300	-0.44688400	-0.03523000
C	-0.86286200	-1.60335800	0.11418000
C	0.55189500	-1.59912900	0.08260500
C	1.32131900	-0.45856300	-0.09462500
C	0.58290300	0.76871800	-0.24863900
C	-0.87177200	0.77262900	-0.22606500
C	2.79541200	-0.52182300	-0.12437700
C	3.44708900	-1.66054600	-0.63953600
C	4.82933800	-1.75148900	-0.64208500
C	5.63829900	-0.71218700	-0.13157500
C	4.98531800	0.43228600	0.37663000
C	3.60134200	0.52316900	0.37387500
C	7.07431200	-0.91306200	-0.18340800
N	-1.41188000	1.97997400	-0.40093400
N	1.10427000	1.98564100	-0.44650400
S	-0.15984900	3.02774000	-0.58179000
C	-10.58426600	0.99993700	0.03065500
O	9.63607000	-1.80171700	-0.53534600
C	9.52923400	-0.58387800	0.05914500
C	8.11963400	-0.13881400	0.23107000
O	10.49748500	0.05790300	0.40620000
C	7.98588700	1.13971800	0.85742600
N	7.86665100	2.17890600	1.36764600
C	-10.65132000	-1.47892200	0.43020800
H	-8.34697200	-2.45292700	0.48253300
H	-5.91169900	-2.51290800	0.41863300
H	-5.79720100	1.72668000	-0.27943000
H	-8.23418800	1.81627700	-0.22294900
H	-1.37865200	-2.54524800	0.27057700
H	1.05631000	-2.54813800	0.23745400
H	2.86185400	-2.46661200	-1.07100200
H	5.30488800	-2.63736500	-1.05599800
H	5.55990300	1.25671500	0.77962300
H	3.13157600	1.41621900	0.76594000
H	7.37316200	-1.85578900	-0.63349400
H	-11.66062300	0.82642800	0.07138900
H	-10.35902100	1.49061500	-0.92573800
H	-10.32048700	1.69598400	0.84013300
H	10.59302200	-1.97845400	-0.59135500
H	-11.71690600	-1.24743800	0.39387300
H	-10.42625100	-1.89316400	1.42388600
H	-10.45169300	-2.25947100	-0.31610900

LS-387-1A

N	-9.70013000	-0.13253300	0.15183200
C	-8.32400600	-0.13746300	0.11141800
C	-7.59920900	-1.34047600	0.26073800
C	-6.21136200	-1.34406300	0.22011900
C	-5.47774100	-0.14369900	0.02890600
C	-6.21234400	1.05206400	-0.11914300
C	-7.59257700	1.06763400	-0.08141800
C	-4.06767000	-0.18562100	-0.00470600
C	-2.84850200	-0.27312300	-0.02612600
C	-1.43963500	-0.31263800	-0.05693400
C	-0.70553400	-1.48516400	0.07039200
C	0.70883400	-1.50462600	0.04113400
C	1.49857500	-0.37448800	-0.11224900
C	0.78176700	0.86817400	-0.24297600
C	-0.67236200	0.89755700	-0.22235700
C	2.97134600	-0.46305000	-0.14123500
C	3.60367700	-1.60271600	-0.67777800
C	4.98415200	-1.71801700	-0.67980600
C	5.81023300	-0.70333800	-0.14779100
C	5.17663500	0.44219900	0.38187600
C	3.79442800	0.55751200	0.37890000
C	7.24285200	-0.92847300	-0.20133700
N	-1.19167500	2.11670200	-0.37387400
N	1.32376400	2.07980800	-0.41603700
S	0.07848600	3.14627700	-0.53228600
C	-10.43383000	1.11455500	0.00740000
O	9.78951300	-1.85565600	-0.56354400
C	9.70286900	-0.64546900	0.04919900
C	8.30056900	-0.17923700	0.22682800
O	10.68112600	-0.02516700	0.40679000
C	8.18787400	1.09129600	0.87332800
N	8.08537000	2.12410600	1.39983900
C	-10.42787700	-1.37661100	0.34179200
O	-5.57883400	-2.53627400	0.37094000
H	-8.09495000	-2.29015600	0.41238000
H	-5.66610700	1.97859500	-0.26668500
H	-8.10909800	2.01113200	-0.20210800
H	-1.23448300	-2.42303800	0.20733500
H	1.19593500	-2.46529400	0.17787000
H	3.00534000	-2.38975500	-1.12610400
H	5.44473200	-2.60390400	-1.11024100
H	5.76478900	1.24823900	0.80208500
H	3.33990300	1.45071300	0.78825900
H	7.52587100	-1.86871700	-0.66656500
H	-11.50282500	0.91104200	0.08468500
H	-10.24878200	1.58795100	-0.96678400
H	-10.16938000	1.83592800	0.79250000
H	10.74332300	-2.04802100	-0.62194100
H	-11.49921300	-1.17101600	0.33068500

H	-10.18213100	-1.84991600	1.30256000
H	-10.21219900	-2.09874000	-0.45751800
H	-4.61921100	-2.37161000	0.32130500

LS-387-1B

N	-9.69252800	-0.00776500	0.07647400
C	-8.31465500	-0.07770800	0.07848500
C	-7.65033100	-1.31325800	0.22194300
C	-6.25539200	-1.40388100	0.21591500
C	-5.47127100	-0.22349400	0.05812400
C	-6.14797500	1.00749500	-0.07650300
C	-7.52524300	1.09575100	-0.06790300
C	-4.06267500	-0.28742700	0.03141700
C	-2.84245900	-0.35128600	0.00094700
C	-1.43483400	-0.37498100	-0.03142300
C	-0.68571600	-1.53807500	0.10571900
C	0.72785200	-1.54206600	0.07264200
C	1.50638300	-0.40512800	-0.09385300
C	0.77561300	0.82845000	-0.23389000
C	-0.67927300	0.84168400	-0.21031500
C	2.97926000	-0.47935200	-0.12613600
C	3.62241600	-1.62347600	-0.64116200
C	5.00370900	-1.72476200	-0.64622000
C	5.82186600	-0.69066500	-0.13935800
C	5.17793400	0.45929400	0.36840200
C	3.79479200	0.56026000	0.36879300
C	7.25575500	-0.90202200	-0.19441000
N	-1.21207200	2.05345300	-0.37267000
N	1.30372300	2.04434800	-0.41959600
S	0.04594800	3.09605700	-0.54339500
C	-10.35762300	1.28416600	0.01769400
O	9.80994300	-1.81131500	-0.55002300
C	9.71372900	-0.58971600	0.03905300
C	8.30833700	-0.13355700	0.21338500
O	10.68818200	0.04626700	0.37971100
C	8.18594200	1.14885700	0.83395000
N	8.07608000	2.19142500	1.33942600
C	-10.48280000	-1.20465200	0.30638000
N	-5.63187500	-2.62490800	0.41138600
H	-8.21644700	-2.22776900	0.35629100
H	-5.55217500	1.90759700	-0.19405700
H	-7.99133500	2.06670900	-0.17384200
H	-1.20472400	-2.47940800	0.25589700
H	1.22565000	-2.49599400	0.21850900
H	3.03105700	-2.42584500	-1.07111400
H	5.47175800	-2.61477000	-1.05992300
H	5.75933000	1.28017800	0.76892600
H	3.33271400	1.45737300	0.76054900
H	7.54644800	-1.84880300	-0.64135300
H	-11.43777100	1.12982300	0.01391000
H	-10.09439000	1.82993300	-0.89744900

H	-10.10656100	1.92113300	0.87822800
H	10.76549400	-1.99474600	-0.60806800
H	-11.54276000	-0.95173900	0.25128700
H	-10.28782600	-1.64856000	1.29400100
H	-10.28099400	-1.96969100	-0.45512600
H	-4.65957500	-2.66427600	0.13243100
H	-6.16141500	-3.44625800	0.15252700

LS-387-1C

N	-9.51821300	0.28405500	0.31844000
C	-8.14814200	0.16449600	0.22448200
C	-7.51596800	-1.09743400	0.32444400
C	-6.13749200	-1.22523400	0.23020900
C	-5.30686500	-0.09444300	0.03349500
C	-5.94635600	1.15960700	-0.07553200
C	-7.31984500	1.29667100	0.01468600
C	-3.89627400	-0.18771800	-0.00221100
C	-2.67747600	-0.21710800	-0.03657500
C	-1.26802900	-0.25456600	-0.05901500
C	-0.53868800	-1.41563200	0.16357700
C	0.87598900	-1.44155800	0.14421500
C	1.67028900	-0.32890700	-0.09188300
C	0.95851900	0.90192200	-0.32349400
C	-0.49588800	0.93637900	-0.31392300
C	3.14297700	-0.42318700	-0.10276600
C	3.77595800	-1.60315500	-0.54293700
C	5.15601100	-1.72178200	-0.52517200
C	5.98132000	-0.67064300	-0.06794000
C	5.34727100	0.51483100	0.36462600
C	3.96554900	0.63314300	0.34146400
C	7.41334500	-0.90303500	-0.09215500
N	-1.00992200	2.14194400	-0.56362500
N	1.50606000	2.09469500	-0.58706800
S	0.26470800	3.15229000	-0.79371000
C	-10.13728800	1.59922600	0.29043100
O	9.95999900	-1.86051400	-0.36606200
C	9.87201000	-0.60982400	0.15923900
C	8.47016000	-0.12757000	0.28946800
O	10.84949200	0.03061000	0.48201400
C	8.35643500	1.18698800	0.84074000
N	8.25350300	2.25583900	1.28952500
C	-10.32879300	-0.88022700	0.63620800
O	-5.60374100	-2.48221900	0.38877000
C	-5.09718300	-3.08785200	-0.80259200
H	-8.08287300	-2.00480800	0.48834300
H	-5.32846400	2.04031800	-0.22183500
H	-7.75074600	2.28679400	-0.06576900
H	-1.07501700	-2.33526100	0.37397800
H	1.35928700	-2.38958900	0.36023400
H	3.17864400	-2.42149500	-0.93263300
H	5.61714400	-2.63991300	-0.88107200

H	5.93486400	1.35005100	0.72431700
H	3.51057200	1.55707000	0.67509800
H	7.69703300	-1.87572200	-0.48461800
H	-11.22074100	1.48603200	0.35205200
H	-9.90895300	2.12866400	-0.64348800
H	-9.81111700	2.23177600	1.12920600
H	10.91363000	-2.05935100	-0.40059000
H	-11.37923000	-0.58726600	0.66907800
H	-10.06382500	-1.31659300	1.61025700
H	-10.22261100	-1.66255900	-0.12661100
H	-4.72338700	-4.07080200	-0.50693500
H	-4.27971100	-2.50263800	-1.23759200
H	-5.89510000	-3.21042200	-1.54728100

LS-387-3D

N	-10.24097000	-0.29356100	0.00410600
C	-8.86312700	-0.32509800	0.00826900
C	-8.09890100	0.86935300	-0.01295100
C	-6.71510000	0.83323300	-0.00895700
C	-6.01099200	-0.38959700	0.01637000
C	-6.76968900	-1.57861200	0.03773100
C	-8.15411200	-1.55288000	0.03388500
C	-4.59574500	-0.41415200	0.01924200
C	-3.37623700	-0.41242100	0.02033300
C	-1.96676300	-0.41138700	0.02017300
C	-1.20998700	-1.57637700	0.04765100
C	0.20436100	-1.56543600	0.04667100
C	0.97274800	-0.41006800	0.02135600
C	0.23221100	0.82546600	0.00249300
C	-1.22242800	0.82366200	-0.00586900
C	2.44676800	-0.46648900	0.01079500
C	3.11333000	-1.55335100	-0.59146400
C	4.49487400	-1.65369300	-0.57458800
C	5.28611300	-0.64965000	0.02110800
C	4.62560000	0.43762800	0.62582400
C	3.24104700	0.52593300	0.61946900
C	6.75733300	-0.78062200	0.02297200
N	-1.76425700	2.04208100	-0.04435200
N	0.75159400	2.05853500	-0.03336300
S	-0.51408500	3.10699400	-0.06901800
C	-10.99982100	-1.53295000	0.02197100
O	9.57905500	-1.07861300	0.45126400
C	9.15333800	0.13220400	0.04346700
C	7.66182600	0.25689200	-0.01254900
O	9.90027900	1.04402000	-0.23656600
C	7.24164000	1.61321500	-0.18720100
N	6.92838400	2.72412600	-0.33329800
C	-10.94198700	0.97948400	-0.02032500
C	7.21173800	-2.14636400	0.02550700
N	7.44311200	-3.28596000	-0.00029200
H	-8.59256500	1.83334000	-0.03349300

H	-6.15400500	1.76261700	-0.02601200
H	-6.25394200	-2.53410200	0.05758500
H	-8.69131400	-2.49339800	0.05117200
H	-1.72456200	-2.53121700	0.07918700
H	0.70862200	-2.52554800	0.09801300
H	2.54003500	-2.31926100	-1.10380500
H	4.97072000	-2.50686100	-1.04676600
H	5.19178100	1.20861600	1.13310700
H	2.76574000	1.37135800	1.09979400
H	-12.06571300	-1.30078900	0.01083600
H	-10.79212600	-2.12633400	0.92341200
H	-10.78056000	-2.15848000	-0.85458100
H	10.55404400	-1.05280200	0.42027700
H	-12.01747400	0.79646000	-0.01583400
H	-10.70138500	1.56157700	-0.92095200
H	-10.69989100	1.59557100	0.85685300
LS-387-3E			
N	10.13210100	-0.25909500	-0.06673800
C	8.75433400	-0.30396300	-0.06137400
C	8.05639900	-1.53311100	-0.17338400
C	6.67211600	-1.57226900	-0.16648000
C	5.90280800	-0.39603200	-0.04872800
C	6.59584100	0.82819800	0.06230700
C	7.97934200	0.87770500	0.05676500
C	4.48747500	-0.43311100	-0.04077700
C	3.26818200	-0.43955300	-0.03069800
C	1.85827800	-0.44303100	-0.01825600
C	1.10321300	-1.60467700	-0.11843400
C	-0.31172000	-1.59650000	-0.10620900
C	-1.08082400	-0.44710600	0.00227200
C	-0.34207000	0.78547600	0.10124800
C	1.11258600	0.78593500	0.09820800
C	-2.55550500	-0.50592800	0.01784600
C	-3.21742200	-1.62315500	0.56651300
C	-4.59920700	-1.71948200	0.55924200
C	-5.39285300	-0.68740700	0.01471400
C	-4.73820600	0.43309700	-0.53423200
C	-3.35305900	0.51883500	-0.52844200
C	-6.84410000	-0.85422100	0.03628200
N	1.65292800	1.99999500	0.21654700
N	-0.86320500	2.01205000	0.22672200
S	0.40118200	3.05791400	0.32457800
C	10.82125100	1.01590500	0.04158800
O	-9.62979300	-1.56094400	-0.01858300
C	-9.31840000	-0.24766300	-0.09418600
C	-7.85803100	0.05809500	-0.09756900
O	-10.16506200	0.61916900	-0.16302700
C	-7.58269500	1.45345500	-0.22975800
N	-7.36913000	2.59209600	-0.33877700
C	10.90218600	-1.48619000	-0.18034000

F	-7.20062600	-2.12758400	0.25046900
H	8.60168600	-2.46439600	-0.26735800
H	6.16514300	-2.52866300	-0.25414700
H	6.02636700	1.74801600	0.15459400
H	8.46401900	1.84229400	0.14658100
H	1.61888200	-2.55422500	-0.21861500
H	-0.81566200	-2.55172200	-0.21827600
H	-2.63942200	-2.41468100	1.03299800
H	-5.07567200	-2.58998300	0.99408200
H	-5.30469600	1.23780100	-0.98335600
H	-2.88019100	1.39200800	-0.95898600
H	11.89824900	0.84515000	0.00757600
H	10.58923600	1.52780200	0.98614200
H	10.55978400	1.69222100	-0.78409500
H	-10.60534500	-1.58497300	-0.03458700
H	11.96594500	-1.24582000	-0.15101700
H	10.69970600	-2.01102400	-1.12467300
H	10.68916400	-2.17908900	0.64556000
LS-387-3F			
N	10.79547400	-0.45067500	0.03786300
C	9.41646100	-0.43329500	0.02967000
C	8.66460900	-1.63501900	0.04002000
C	7.27979000	-1.61203400	0.03143500
C	6.56338400	-0.39745400	0.01218100
C	7.31026000	0.79944400	0.00198600
C	8.69497800	0.78696600	0.01050300
C	5.14707000	-0.37425900	0.00356000
C	3.92862100	-0.33563900	-0.00375600
C	2.51819100	-0.29797800	-0.01197700
C	1.73347900	-1.44338600	0.00003300
C	0.31828100	-1.39879200	-0.00980600
C	-0.42007900	-0.22584200	-0.03279000
C	0.35028100	0.99049300	-0.05492400
C	1.80469900	0.95484200	-0.03759600
C	-1.89771000	-0.24325600	-0.02778000
C	-2.59253700	-1.26678900	0.64649000
C	-3.97788900	-1.33514000	0.62984800
C	-4.73542100	-0.36048000	-0.04646700
C	-4.04925900	0.66456800	-0.71844600
C	-2.66078000	0.72357200	-0.70783300
C	-6.21213600	-0.42798300	-0.04468300
N	2.37496600	2.16102700	-0.04256800
N	-0.14066700	2.23582600	-0.06991900
S	1.14914900	3.25467800	-0.06641200
C	11.54102300	0.79658700	0.02953600
O	-9.06267500	-0.06355500	1.13848100
C	-8.54591900	0.61389000	0.09623900
C	-7.02885900	0.63887700	0.14373100
O	-9.19658400	1.19550300	-0.73558100
C	-6.52984600	1.95332200	0.40955800

N	-6.20029500	3.04293800	0.65147500
C	11.50895200	-1.71616500	0.05865500
C	-6.81293100	-1.81410200	-0.25419900
F	-8.05001800	-1.77197300	-0.78868000
F	-6.89684000	-2.48532900	0.91719700
F	-6.05368900	-2.54969900	-1.08558700
H	9.16785600	-2.59422000	0.05512000
H	6.73079800	-2.54903900	0.03982100
H	6.78260100	1.74828200	-0.01277100
H	9.22250600	1.73311800	0.00213500
H	2.22479800	-2.41098600	0.00899000
H	-0.20999600	-2.34735500	-0.02714700
H	-2.03858800	-2.00518800	1.21768600
H	-4.47716000	-2.13195400	1.17043900
H	-4.60292000	1.41097500	-1.27759800
H	-2.16166600	1.52417900	-1.23879200
H	12.60940100	0.57559600	0.03876500
H	11.31543700	1.41371000	0.91058900
H	11.32701000	1.39465800	-0.86735900
H	-10.03446500	-0.02382000	1.04373900
H	12.58265100	-1.52245300	0.06062500
H	11.27773900	-2.33026800	-0.82310200
H	11.26998100	-2.30616400	0.95479100

LS-387-4D

N	10.16868200	-0.14920000	-0.10294100
C	8.79341900	-0.23225400	-0.06548400
C	8.13328400	-1.48181400	0.05197600
C	6.75108700	-1.55994400	0.08158800
C	5.94647700	-0.40447400	-0.00152800
C	6.60173000	0.84032300	-0.11445900
C	7.98281200	0.92878600	-0.14482000
C	4.53327100	-0.48113800	0.02636200
C	3.31466000	-0.52122000	0.04619100
C	1.90576200	-0.56142700	0.06651100
C	1.18323300	-1.74285000	0.17769800
C	-0.23142200	-1.77109700	0.19603400
C	-1.03060000	-0.64125400	0.10723500
C	-0.32697100	0.61013000	0.00175600
C	1.12625000	0.64853000	-0.02566900
C	-2.50462500	-0.73408800	0.11282400
C	-3.15365600	-1.86848200	-0.41298300
C	-4.53575400	-1.98691100	-0.38364700
C	-5.35239500	-0.96565900	0.12938500
C	-4.70624700	0.16577400	0.68854200
C	-3.31003300	0.27177800	0.67143800
C	-6.80411200	-1.15551500	0.07369200
N	1.63098000	1.87771100	-0.14557600
N	-0.88326600	1.82315700	-0.10135200
S	0.35048000	2.90310900	-0.21740700
C	10.81921600	1.14827400	-0.18272500

O	-9.43710700	-1.87317300	0.17906200
C	-9.20540200	-0.63715400	-0.33031300
C	-7.75522100	-0.27520300	-0.32773400
O	-10.08679000	0.08316400	-0.74034400
C	-7.45873600	1.01605800	-0.87234400
N	-7.21086300	2.04115600	-1.36123400
C	10.97588400	-1.35188000	0.01623400
C	-5.43802700	1.17634500	1.39764000
N	-5.99257900	1.98314400	2.02531600
H	8.70668400	-2.39829300	0.12077700
H	6.27353400	-2.53113200	0.17167300
H	6.00449300	1.74482000	-0.17810100
H	8.43763500	1.90805400	-0.23160200
H	1.72509600	-2.67919500	0.26186700
H	-0.70695700	-2.74018000	0.31433600
H	-2.56834000	-2.65768900	-0.87382600
H	-5.00455800	-2.87159600	-0.80594900
H	-2.85234400	1.14771000	1.11141600
H	-7.15835400	-2.14974900	0.33381400
H	11.90057700	1.00598900	-0.20715000
H	10.57914700	1.78318700	0.68210200
H	10.53010300	1.69087700	-1.09296700
H	-10.40195200	-2.00347000	0.12598700
H	12.03118900	-1.08324200	-0.04955000
H	10.75920500	-2.06577200	-0.78993200
H	10.81435500	-1.86355600	0.97597900
LS-387-4E			
N	-10.08228400	-0.19733800	-0.04062700
C	-8.70530500	-0.26367700	-0.01965400
C	-8.02824400	-1.50693800	0.06009800
C	-6.64479000	-1.56804000	0.07153900
C	-5.85565000	-0.40084300	0.00734300
C	-6.52785500	0.83767700	-0.06718800
C	-7.91034400	0.90910200	-0.07920600
C	-4.44113200	-0.46052600	0.01744500
C	-3.22206200	-0.48671300	0.02285400
C	-1.81244700	-0.51366600	0.02695900
C	-1.07800200	-1.69029500	0.10168000
C	0.33671700	-1.70588300	0.10421300
C	1.12684400	-0.56774300	0.03420300
C	0.40980700	0.67983700	-0.03307200
C	-1.04466200	0.70496800	-0.04400600
C	2.60061100	-0.65247100	0.02610200
C	3.24946700	-1.78611700	-0.51109100
C	4.62894500	-1.89488000	-0.48945800
C	5.45432400	-0.87208100	0.02899100
C	4.78339700	0.23456200	0.57948100
C	3.40422800	0.35941200	0.57891800
C	6.89483900	-1.06478500	-0.00492600
N	-1.56327700	1.93125600	-0.12800700

N	0.95203200	1.90106900	-0.11192600
S	-0.29348200	2.97094900	-0.18822900
C	-10.74955000	1.09370100	-0.06625200
O	9.46125200	-1.97350500	0.05822500
C	9.33719200	-0.62872400	-0.09679100
C	7.91747500	-0.16957500	-0.09541200
O	10.29449500	0.10342900	-0.21838300
C	7.76071400	1.23841000	-0.29622200
N	7.65457500	2.37567300	-0.51351400
C	-10.87281600	-1.41192400	0.06799600
F	5.49368100	1.19597200	1.19116300
H	-8.58928800	-2.43201300	0.11422800
H	-6.15409800	-2.53492700	0.13268400
H	-5.94289800	1.75113300	-0.11521000
H	-8.37838900	1.88433900	-0.13614100
H	-1.61032500	-2.63352800	0.16877800
H	0.82174900	-2.67290100	0.19455700
H	2.66487100	-2.57424000	-0.97327600
H	5.10277300	-2.77578100	-0.91466100
H	2.96870100	1.24569800	1.02029500
H	7.20358500	-2.10672700	0.00237300
H	-11.82933100	0.93862100	-0.08380500
H	-10.47812500	1.67138600	-0.96011000
H	-10.50724100	1.70145600	0.81750100
H	10.41980900	-2.14926000	0.03486900
H	-11.93198300	-1.15626300	0.01274700
H	-10.69813700	-1.93594000	1.01899800
H	-10.65258900	-2.11073200	-0.75010300

LS-387-4F

N	10.57150900	-0.51468600	0.12714800
C	9.19353800	-0.50392800	0.12365100
C	8.44680800	-1.63815800	0.53243300
C	7.06239900	-1.62207100	0.52688400
C	6.34083800	-0.48158600	0.11609600
C	7.08229200	0.64806800	-0.29107800
C	8.46655600	0.64183900	-0.28901100
C	4.92559200	-0.46529100	0.11056800
C	3.70649300	-0.43396100	0.09959900
C	2.29754900	-0.40371100	0.08993600
C	1.51654800	-1.48248200	0.48616400
C	0.10291400	-1.44306300	0.47611600
C	-0.64141700	-0.34160700	0.07763100
C	0.12449200	0.80670300	-0.33428800
C	1.57887000	0.77244000	-0.33459900
C	-2.11625000	-0.37080300	0.08242800
C	-2.81020200	-1.57545500	-0.12757600
C	-4.19532200	-1.63055300	-0.08780200
C	-4.97400400	-0.48479800	0.16425400
C	-4.27265500	0.73491600	0.37424700
C	-2.88617500	0.78465400	0.32094700

C	-6.43022300	-0.52430200	0.24386500
N	2.14579000	1.90305900	-0.75865700
N	-0.36868400	1.97394900	-0.76554500
S	0.91888100	2.92982200	-1.12867800
C	11.31171600	0.66029900	-0.30224900
O	-9.09619300	-0.18598100	0.70457800
C	-8.78051000	-1.29654100	-0.00656300
C	-7.31238200	-1.45365100	-0.22071400
O	-9.61509700	-2.07241600	-0.41907900
C	-6.96116900	-2.62653400	-0.96166200
N	-6.67051500	-3.58046300	-1.56123400
C	11.29106800	-1.70116600	0.55949700
C	-5.01689700	2.02522700	0.63968600
F	-5.72276900	1.96556800	1.79924900
F	-4.19316200	3.08473100	0.74040500
F	-5.90688900	2.30229100	-0.34110700
H	8.95439400	-2.53888100	0.85575000
H	6.51728500	-2.50581900	0.84496500
H	6.55026900	1.53857200	-0.61174700
H	8.98995600	1.53433800	-0.60989400
H	2.01086100	-2.38708800	0.82487200
H	-0.42013500	-2.32550300	0.83163800
H	-2.26063400	-2.48390400	-0.35210600
H	-4.68007800	-2.58409900	-0.25139500
H	-2.38829000	1.73296400	0.46186000
H	-6.89968900	0.31237800	0.74460600
H	12.38100200	0.45605300	-0.23130600
H	11.09131200	1.53414000	0.32663400
H	11.08614100	0.92600200	-1.34438500
H	-10.06864400	-0.18348100	0.77226200
H	12.36377400	-1.51407100	0.49310600
H	11.05956200	-2.57145600	-0.07039200
H	11.05701800	-1.96337000	1.60071900
LS-387-5D			
N	-10.02482400	-0.26836300	0.16489100
C	-8.64720700	-0.32279100	0.13775800
C	-7.94952700	-1.50801100	0.48281000
C	-6.56622200	-1.55987500	0.44579600
C	-5.79754900	-0.43951000	0.06754700
C	-6.48986600	0.74213500	-0.27246700
C	-7.87244900	0.80393800	-0.23796000
C	-4.38311400	-0.48917500	0.03284400
C	-3.16453200	-0.50911800	-0.00265000
C	-1.75509200	-0.52953100	-0.04210300
C	-1.00876800	-1.65313700	0.29375800
C	0.40753500	-1.66471800	0.26045800
C	1.16884600	-0.56391200	-0.09261200
C	0.44430400	0.61345700	-0.47703500
C	-1.00626000	0.63489300	-0.44843200
C	2.64521800	-0.62275800	-0.11367500

C	3.28764600	-1.66464400	-0.80015000
C	4.67083600	-1.75460400	-0.84818000
C	5.49306000	-0.80372700	-0.20799000
C	4.86120600	0.23373800	0.50349300
C	3.46666400	0.32336700	0.55532400
C	6.93160100	-0.98526700	-0.33396700
N	-1.53299100	1.79422900	-0.84816100
N	0.98413800	1.75425200	-0.91871000
S	-0.26797700	2.76915300	-1.23429400
C	-10.70977500	0.98171200	-0.12036300
O	9.48038400	-1.79747800	-0.88682500
C	9.39194700	-0.67287700	-0.13129600
C	7.98408500	-0.26723000	0.14790800
O	10.36515900	-0.07213000	0.26728300
C	7.86586800	0.91374900	0.94580100
N	7.75703800	1.87302300	1.59519500
C	-10.78957400	-1.41311700	0.63112900
C	2.89540800	1.35302200	1.37768500
N	2.46478700	2.16946000	2.08455200
H	-8.49413700	-2.39482300	0.78347400
H	-6.05950000	-2.48157500	0.71612400
H	-5.92053700	1.61973100	-0.56320700
H	-8.35634900	1.73616900	-0.50284300
H	-1.53159000	-2.55158400	0.60481900
H	0.91647800	-2.57652100	0.56131700
H	2.68139200	-2.39432000	-1.32767200
H	5.13334500	-2.56753500	-1.40198200
H	5.44286800	0.97512600	1.03610900
H	7.21608000	-1.85336700	-0.92175900
H	-11.78735200	0.82068400	-0.06344600
H	-10.47780800	1.34501500	-1.13005000
H	-10.44429800	1.77333800	0.59555000
H	10.43456700	-1.96082600	-1.00242500
H	-11.85396800	-1.18079700	0.57233900
H	-10.55386800	-1.67293000	1.67360200
H	-10.60478200	-2.29962100	0.01012800
LS-387-5E			
N	-9.98512700	-0.25792600	0.20499600
C	-8.60722300	-0.30857500	0.16325100
C	-7.90619000	-1.51542800	0.41192800
C	-6.52288200	-1.56253100	0.36107300
C	-5.75758700	-0.41586400	0.06433500
C	-6.45337900	0.78691000	-0.18003900
C	-7.83603000	0.84403500	-0.13169600
C	-4.34270300	-0.46087300	0.01698500
C	-3.12454200	-0.47662400	-0.02691900
C	-1.71437400	-0.49302800	-0.07390100
C	-0.96704000	-1.64301000	0.14636200
C	0.44999200	-1.65031500	0.10736600
C	1.21133200	-0.52207500	-0.14052400

C	0.48535300	0.68933500	-0.39835400
C	-0.96638500	0.70627200	-0.36451800
C	2.68605000	-0.58240000	-0.17705900
C	3.35330500	-1.57752700	-0.91189800
C	4.73878800	-1.65139100	-0.93927800
C	5.53360400	-0.72952600	-0.22463100
C	4.87782500	0.26608400	0.52884200
C	3.49586000	0.31539800	0.54055400
C	6.97475100	-0.88874500	-0.32494200
N	-1.49712500	1.89847000	-0.64393400
N	1.02191300	1.87104400	-0.72065900
S	-0.23354800	2.90892500	-0.93628400
C	-10.67317200	1.00831600	0.01647200
O	9.54966100	-1.62541200	-0.87126000
C	9.42475300	-0.57069100	-0.02436800
C	8.00634100	-0.20213200	0.24371800
O	10.38065200	-0.00019700	0.45438300
C	7.85128600	0.90749100	1.13263800
N	7.71329900	1.80835200	1.85613400
C	-10.74574600	-1.43698100	0.58350300
F	2.90816200	1.24603300	1.31086200
H	-8.44765700	-2.42346700	0.64794400
H	-6.01368600	-2.50163700	0.55657000
H	-5.88700000	1.68483200	-0.40772800
H	-8.32244600	1.79277600	-0.32324300
H	-1.48846200	-2.56864800	0.36739200
H	0.95992800	-2.58750000	0.31327400
H	2.76291400	-2.28181400	-1.49002400
H	5.22265500	-2.42436300	-1.52986500
H	5.41931300	0.99504500	1.11753500
H	7.28479600	-1.70582100	-0.97003400
H	-11.75011800	0.84242800	0.07179300
H	-10.45106100	1.44413600	-0.96655800
H	-10.40166200	1.74675900	0.78509000
H	10.50899100	-1.76915500	-0.96791800
H	-11.81058100	-1.20092800	0.55462000
H	-10.49995300	-1.78083200	1.59895800
H	-10.56779300	-2.27039400	-0.10917000

LS-387-5F

N	-10.26379200	-0.30826600	0.15428500
C	-8.88601500	-0.36483600	0.13378600
C	-8.18382000	-1.43959100	0.73505300
C	-6.79985800	-1.49104300	0.71248300
C	-6.03556800	-0.48073900	0.09325700
C	-6.73278800	0.58945500	-0.50586600
C	-8.11611200	0.64977400	-0.48902600
C	-4.61996300	-0.53150700	0.06975200
C	-3.40174600	-0.55571400	0.03831300
C	-1.99081100	-0.58011200	0.00056400
C	-1.23988300	-1.60943500	0.55174700

C	0.17922800	-1.62515200	0.51442200
C	0.93298300	-0.61620700	-0.05161600
C	0.20455100	0.45677800	-0.66185900
C	-1.24670400	0.48072200	-0.63476800
C	2.41710600	-0.69242600	-0.09470600
C	2.98768700	-1.78815200	-0.76518200
C	4.36109800	-1.94344900	-0.86992800
C	5.24287900	-1.00446400	-0.30025800
C	4.67805000	0.07938100	0.39862400
C	3.29795200	0.23692600	0.50681400
C	6.66774300	-1.24578500	-0.47211700
N	-1.77676700	1.53587900	-1.25607600
N	0.74223000	1.48862100	-1.31980200
S	-0.51331300	2.41679200	-1.83268700
C	-10.95783300	0.80332000	-0.47366700
O	9.16136400	-2.18298400	-1.07704200
C	9.14346000	-1.01289300	-0.38826600
C	7.76382300	-0.54034100	-0.07649000
O	10.15339800	-0.42598400	-0.06756400
C	7.72180900	0.68964600	0.65244000
N	7.68038300	1.68967500	1.24570600
C	-11.02776100	-1.36237800	0.79951100
C	2.82676500	1.43462500	1.31209200
F	2.74447600	2.54939300	0.56039300
F	3.69441200	1.70349500	2.31482200
F	1.62046800	1.23676200	1.88353000
H	-8.72522200	-2.24029000	1.22408300
H	-6.28950900	-2.32685900	1.18193800
H	-6.16719900	1.38034100	-0.98911500
H	-8.60425000	1.49253600	-0.96321600
H	-1.75810900	-2.43054500	1.03625900
H	0.69221100	-2.46151300	0.98165800
H	2.32631700	-2.51240200	-1.23012500
H	4.76437400	-2.79794500	-1.40737200
H	5.31722700	0.80304900	0.88665800
H	6.89729300	-2.15482600	-1.02087800
H	-12.03386600	0.66657400	-0.35614800
H	-10.74016400	0.86598800	-1.54913000
H	-10.68715500	1.76520500	-0.01607300
H	10.10376100	-2.38658200	-1.22156700
H	-12.09183000	-1.13634100	0.71611700
H	-10.78319300	-1.44880000	1.86751700
H	-10.85201200	-2.34116200	0.33107600

LS-387-12A

N	-9.45856000	-0.27287500	0.05938000
C	-8.06099500	-0.27609600	0.07936800
C	-7.32860100	-1.46330800	0.23890800
C	-5.93595000	-1.48546100	0.19910000
C	-5.20976200	-0.29020300	0.00899900
C	-5.94174700	0.90901400	-0.15615100

C	-7.32214700	0.92764000	-0.13964600
C	-3.79869200	-0.32130000	-0.01953400
C	-2.57895900	-0.39110500	-0.03585600
C	-1.16904700	-0.41048500	-0.06244400
C	-0.41882700	-1.57017000	0.08313300
C	0.99604600	-1.56945000	0.05818400
C	1.76884300	-0.42984900	-0.10906000
C	1.03412200	0.80007400	-0.25976100
C	-0.42016700	0.80864100	-0.24306400
C	3.24311200	-0.49602200	-0.13275600
C	3.89430500	-1.63171100	-0.65453600
C	5.27655500	-1.72517300	-0.65181900
C	6.08486800	-0.69155800	-0.12934600
C	5.43210500	0.44959100	0.38601600
C	4.04820400	0.54305300	0.37826500
C	7.52144100	-0.89436400	-0.17689100
N	-0.95677700	2.01790600	-0.41354000
N	1.55902700	2.01672400	-0.44924900
S	0.29930900	3.06318600	-0.58403400
C	-10.17870000	0.66694100	0.92132900
O	10.08378800	-1.78386800	-0.52142800
C	9.97591300	-0.56834700	0.07670900
C	8.56535200	-0.12297600	0.24499900
O	10.94241700	0.07229100	0.42999200
C	8.43006700	1.15327200	0.87582400
N	8.30870200	2.19052200	1.38939000
C	-10.13736600	-1.55294800	-0.06020900
O	-5.31504100	-2.68389800	0.37043200
O	-8.02957400	2.08549100	-0.36890000
H	-7.83438200	-2.40273200	0.42176700
H	-5.39129900	1.83081000	-0.32886000
H	-0.93460700	-2.51358600	0.23161300
H	1.49698000	-2.52066600	0.20979600
H	3.30953400	-2.43316000	-1.09521200
H	5.75220600	-2.60849500	-1.07089300
H	6.00633400	1.26939900	0.79869800
H	3.57868800	1.43337900	0.77683500
H	7.82085700	-1.83523900	-0.63040500
H	-10.24380200	0.29558400	1.95807500
H	-11.19687600	0.79226900	0.53781600
H	-9.68821100	1.63757000	0.92072900
H	11.04066500	-1.96202700	-0.57499200
H	-11.18709400	-1.36468500	-0.30647400
H	-10.11106300	-2.15273300	0.86662000
H	-9.69557800	-2.14105500	-0.86905800
H	-4.35376100	-2.52832000	0.33679500
H	-7.39683400	2.80041800	-0.53951200

LS-387-12B

N	-9.44691900	-0.12014500	0.06142600
C	-8.03115400	-0.22730800	0.05711200

C	-7.36569900	-1.44090900	0.24282200
C	-5.96739300	-1.53963800	0.21140500
C	-5.21280100	-0.35905600	-0.00832800
C	-5.89044800	0.86292800	-0.22077700
C	-7.27398000	0.95152600	-0.20344700
C	-3.79987900	-0.40300700	-0.02735600
C	-2.57962500	-0.44557000	-0.03728700
C	-1.17007500	-0.45091600	-0.06322300
C	-0.40951200	-1.60535900	0.07486400
C	1.00493500	-1.59352400	0.04989200
C	1.76957800	-0.44691400	-0.10938400
C	1.02543000	0.77838700	-0.25120700
C	-0.42931700	0.77499800	-0.23517300
C	3.24392500	-0.50258700	-0.13442000
C	3.90305400	-1.63213500	-0.66032300
C	5.28574000	-1.71602500	-0.65944800
C	6.08775400	-0.67853400	-0.13450000
C	5.42732300	0.45665000	0.38473800
C	4.04292400	0.54061700	0.37853000
C	7.52504300	-0.87133200	-0.18408600
N	-0.97487200	1.98135700	-0.39742900
N	1.54112700	2.00034100	-0.43169500
S	0.27257500	3.03785900	-0.55973900
C	-10.00343700	0.62439300	1.19989500
O	10.09273100	-1.74210500	-0.53742800
C	9.97741500	-0.53166400	0.07029100
C	8.56474200	-0.09631400	0.24274500
O	10.94086000	0.11163000	0.42762100
C	8.42190700	1.17413500	0.88339000
N	8.29501200	2.20660900	1.40525300
C	-10.19935900	-1.33659200	-0.21011500
N	-5.33930400	-2.77543800	0.35158900
N	-7.94529200	2.17110800	-0.41494000
H	-7.94144700	-2.34293300	0.42578700
H	-5.29533300	1.74992200	-0.42018100
H	-0.91906600	-2.55343500	0.21446200
H	1.51345100	-2.54200000	0.19401800
H	3.32336200	-2.43612500	-1.10306700
H	5.76711800	-2.59462000	-1.08202800
H	5.99639100	1.27928600	0.79903900
H	3.56752100	1.42675900	0.77935800
H	7.83038600	-1.80714800	-0.64411700
H	-9.95509600	0.04042500	2.13475700
H	-11.05374900	0.86037200	0.99518400
H	-9.45587100	1.55816200	1.33934500
H	11.05078300	-1.91309900	-0.59261900
H	-11.24206200	-1.06565400	-0.40809800
H	-10.19624800	-2.05509700	0.62928000
H	-9.79594800	-1.83399600	-1.09631400
H	-4.36133700	-2.71953000	0.61077000

H	-5.84509200	-3.45472500	0.90585700
H	-7.37849300	2.85068400	-0.91095200
H	-8.83000600	2.02647700	-0.89530700

LS-387-12C

N	-9.05712200	-0.14616600	0.34197900
C	-7.65714600	-0.22739400	0.27597200
C	-6.97717400	-1.45809900	0.33193400
C	-5.58991400	-1.54309900	0.25106200
C	-4.80480200	-0.37739700	0.11750100
C	-5.48365700	0.85742600	0.03383300
C	-6.86447700	0.94053400	0.09048700
C	-3.38948600	-0.42262000	0.08321200
C	-2.17151700	-0.41582500	0.04646000
C	-0.76026100	-0.41262200	0.01353100
C	0.00030100	-1.56084500	0.18740600
C	1.41566100	-1.54905100	0.15312600
C	2.17636700	-0.40772300	-0.05105900
C	1.43018900	0.81145600	-0.22997000
C	-0.02395700	0.80706800	-0.20470100
C	3.65127300	-0.46164200	-0.08489300
C	4.30784300	-1.60321500	-0.58638700
C	5.69086500	-1.68453000	-0.59408200
C	6.49431700	-0.63224900	-0.10239400
C	5.83600400	0.51457000	0.39301200
C	4.45124900	0.59580600	0.39535700
C	7.93210400	-0.82356600	-0.15887800
N	-0.57193700	2.00699400	-0.40566800
N	1.94385800	2.02687500	-0.45548000
S	0.67350100	3.05777400	-0.61100000
C	-9.63846500	0.75970200	1.33890400
O	10.49848100	-1.69791900	-0.51103600
C	10.38575100	-0.47217200	0.06521500
C	8.97319900	-0.03509400	0.23766500
O	11.35022600	0.18263300	0.39753400
C	8.83288000	1.25236600	0.84412600
N	8.70791100	2.29875600	1.33788800
C	-9.81034300	-1.38417100	0.21297300
O	-5.00881300	-2.78307000	0.37800600
C	-4.52895600	-3.35482200	-0.84057300
O	-7.43875500	2.19389900	-0.01531800
C	-8.02663300	2.46220600	-1.29299300
H	-7.51812100	-2.38466900	0.48055700
H	-4.91708500	1.77439000	-0.08913400
H	-0.50991600	-2.50130900	0.36864200
H	1.92650600	-2.49094600	0.32855200
H	3.72608600	-2.41947900	-1.00316000
H	6.17080700	-2.57291700	-0.99728500
H	6.40663000	1.34849600	0.78175000
H	3.97693900	1.49083900	0.77740200
H	8.23555800	-1.77090400	-0.59596900

H	-9.63233600	0.31064400	2.34610000
H	-10.67750800	0.97230800	1.06487700
H	-9.08295900	1.69546700	1.36290000
H	11.45633100	-1.86912600	-0.56940300
H	-10.86467200	-1.13317300	0.05935600
H	-9.74463100	-2.03255100	1.10422800
H	-9.46334200	-1.94994800	-0.65608200
H	-4.11446400	-4.33036300	-0.57626100
H	-3.74578700	-2.73628600	-1.29361100
H	-5.34820200	-3.48982900	-1.55961900
H	-8.43560500	3.47354900	-1.23463900
H	-8.82879600	1.75073700	-1.51879700
H	-7.26682800	2.42116800	-2.08398200
